# Research progress of small molecule protein kinase inhibitors (SMKIs) in the treatment of colorectal cancer: mechanism, application, and future prospects

**DOI:** 10.3389/fphar.2026.1782130

**Published:** 2026-07-17

**Authors:** Lei Liu, Yingjun Chen, Mingxin Guo, Fan Yun, Xifeng Li, Chen Lu, Ying Li, Fuchao Li

**Affiliations:** 1 Department of Gastroenterology, The Affiliated Yixing Hospital of Jiangsu University, Yixing, Jiangsu, China; 2 Department of Geriatrics, Suzhou Research Center of Medical School, Suzhou Hospital, Affiliated Hospital of Medical School, Nanjing University, Suzhou, China; 3 Department of Pharmacy, The Affiliated Yixing Hospital of Jiangsu University, Yixing, China; 4 Department of Cardiothoracic Surgery, Zhongda Hospital, School of Medicine, Southeast University, Nanjing, China; 5 Department of Geriatrics, China Hospital Reform and Development Research Institute of Nanjing University, Nanjing Drum Tower Hospital Nanjing University, Nanjing, China

**Keywords:** colorectal cancer, drug resistance mechanisms, protein kinases, small-molecule kinase inhibitors (SMKIs), targeted therapy

## Abstract

Colorectal cancer (CRC) is among the most common malignancies worldwide, and advanced or metastatic disease remains difficult to treat because of tumor heterogeneity, adaptive resistance, pathway redundancy, drug-related toxicity, and limited predictive biomarkers. Small-molecule kinase inhibitors (SMKIs) provide therapeutic opportunities for selected molecular subgroups by targeting key signaling pathways, but their clinical application is still constrained by complex resistance mechanisms, off-target toxicity, and insufficient biomarker-guided stratification. This narrative review summarizes recent progress in SMKIs for CRC, including molecular targets, clinical evidence, resistance mechanisms, combination strategies, and translational directions. Emerging technologies, including multi-omics profiling, artificial intelligence-assisted drug discovery, patient-derived models, liquid biopsy, molecular imaging, multidrug delivery systems, and adaptive trial designs, can be integrated into a translational “discover–validate–monitor–adapt” workflow and may help address some current limitations in targeted drug development for CRC. Future development of SMKI-based therapy in CRC will require biomarker-guided patient selection, rational combination strategies, dynamic monitoring of resistance, and prospective validation using clinically meaningful endpoints.

## 1 Introduction

Colorectal cancer (CRC) ranks among the most prevalent malignancies globally. As reported in the 2022 global cancer statistics, CRC is responsible for over 1.9 million new cases and approximately 930,000 deaths each year, thereby presenting a substantial public health challenge due to its elevated incidence and mortality rates (Bray et al., 2024). The current standard treatment for early - stage CRC mainly includes curative surgical resection, along with chemotherapy and radiotherapy. In contrast, advanced CRC is predominantly treated with systemic therapies, including chemotherapy, targeted therapy, and immunotherapy (Benson et al., 2024; Wang et al., 2025). However, patients with advanced-stage CRC often face treatment failure. This is due to tumor metastasis, chemotherapy resistance, postoperative recurrence, and severe adverse effects. As a result, their 5-year survival rate is less than 15% (Zeng et al., 2018). Furthermore, conventional chemotherapeutic agents, characterized by their non-specific mechanisms of action, are associated with significant adverse effects such as gastrointestinal toxicity, myelosuppression, and neurotoxicity (Kim, 2015; Guo et al., 2016), highlighting the urgent need for the development of more targeted therapeutic strategies.

In recent years, the introduction of molecular targeted agents, particularly SMKIs, has opened new avenues for the treatment of CRC. Protein kinases play a critical role in regulating key intracellular signaling pathways involved in tumor proliferation, angiogenesis, immune escape, and therapeutic resistance (Attwood et al., 2021). However, their clinical value is highly context dependent. Meaningful benefit is currently most evident in limited biomarker-defined settings, such as *BRAF V600E*-mutant CRC treated with combined BRAF/EGFR blockade, HER2-amplified RAS wild-type CRC treated with HER2-directed strategies, *KRAS G12C*-mutant CRC treated with *KRAS*-targeted combinations, and rare neurotrophic tyrosine receptor kinase (NTRK) fusion-positive tumors (Chong et al., 2022; Bando et al., 2024). For most other SMKIs, reliable predictive biomarkers remain insufficient, and therapeutic efficacy is strongly affected by RAS/BRAF status, HER2 amplification, microsatellite instability/mismatch repair (MSI/MMR) status, tumor sidedness, consensus molecular subtype, rare kinase fusions, and coexisting genomic alterations. Consequently, SMKIs should be regarded as context-dependent therapeutic approaches rather than universally effective precision-medicine agents for all CRC patients.

Despite advancements, current SMKIs encounter several challenges, including resistance mechanisms, off-target toxicities, and the absence of predictive biomarkers. To address these issues, emerging technologies—including multi-omics profiling, AI-assisted drug discovery, patient-derived models, liquid biopsy, molecular imaging, multidrug delivery systems, and adaptive trial design—are being explored (Hugo et al., 2016; You et al., 2022; Lin et al., 2024).

This narrative review provides a comprehensive overview of the molecular mechanisms, clinical applications, resistance mechanisms, and future directions of SMKIs in CRC. It examines key signaling pathways and emerging molecular targets, while clearly distinguishing biological rationale from clinically validated benefit. Finally, we discuss how these emerging technologies may serve as supportive tools within a “discover–validate–monitor–adapt” framework for future biomarker-guided SMKIs development.

## 2 Scope and literature identification strategy

This article constitutes a narrative review derived from a comprehensive literature search across multiple databases, distinguishing it from an informal systematic review or meta-analysis. Its objective is to provide a thorough and critical synthesis of the evidence concerning the mechanisms, clinical efficacy, drug resistance mechanisms, combination therapies, and translational aspects of SMKIs in CRC.

The literature search was independently executed by two authors, Rui Liu and Yingjun Chen, utilizing multiple databases, including PubMed, Web of Science, ClinicalTrials.gov, and pertinent regulatory or guideline sources such as documents from the FDA, EMA, NCCN, ESMO, and CSCO. The search strategy employed a combination of free-text keywords, encompassing but not limited to terms like “colorectal cancer,” “CRC,” “small-molecule kinase inhibitor,” “protein kinase inhibitor,” “targeted therapy,” “EGFR,” “BRAF,” “MEK,” “VEGFR,” “PI3K,” “AKT,” “mTOR,” “Wnt,” “TGF-β,” “JAK/STAT,” “HER2,” “MET,” “drug resistance,” “combination therapy,” “immunotherapy,” “liquid biopsy,” “artificial intelligence,” “allosteric inhibitor,” and “PROTAC.”

Eligibility criteria encompassed original research, clinical trials, translational research, high-quality mechanistic studies, systematic reviews, meta-analyses, significant preclinical studies, as well as regulatory or guideline documents directly pertinent to SMKIs, kinase signaling pathways, resistance mechanisms, or emerging treatment strategies for CRC. Publications not in English, studies conducted on animals or *in vitro*, conference abstracts lacking full text, and abstracts of studies unrelated to CRC or not involving kinase-targeted therapy or associated signaling pathways were excluded. This review does not conform to a systematic review format but intends to present a descriptive synthesis organized by themes from the available evidence.

## 3 Epidemiology and pathogenesis of CRC

CRC is not only the third most commonly diagnosed malignancy worldwide but also the second leading cause of cancer-related death. Despite advances in screening and treatment, the burden of CRC remains substantial, particularly in advanced or metastatic disease (Dekker et al., 2019). CRC risk factors include genetic and environmental elements such as male gender, advanced age, a family history of the disease, obesity, lack of exercise, smoking, and heavy alcohol intake (Murphy and Zaki, 2024).

The development of CRC results from the combined effects of genetic and microenvironmental alterations. About 95% of CRC cases develop from precursor polyps in the intestine, mainly through two routes: the classic adenoma–carcinoma sequence (70%–90%) and the serrated pathway (10%–20%). The former is characterized by stepwise mutations in genes such as *APC*, *KRAS*, and *PIK3CA*, whereas the latter is closely associated with *BRAF* mutations, the CpG island methylator phenotype (Schmitt and Greten, 2021). In addition, epigenetic changes substantially contribute to carcinogenesis, including promoter hypermethylation of tumor suppressor genes (e.g., MLH1), aberrant histone modifications (e.g., loss of H3K27me3), and dysregulation of noncoding RNAs (Tamagawa et al., 2013; Strubberg and Madison, 2017; Feinberg, 2018; Qin et al., 2020). Recent studies have also revealed a key role for the gut microbiota in CRC development (Ocvirk and O’Keefe, 2021; Wang and Fang, 2023).

Clinical data indicate that approximately 25% of patients present with advanced-stage disease with metastases at diagnosis, and an additional ∼20% may develop metachronous metastases after treatment (van der Stok et al., 2017), highlighting the need for mechanism-based therapeutic strategies. In this context, small-molecule protein kinase inhibitors (SMKIs) have attracted increasing attention as pathway-directed agents for selected molecular subgroups of advanced CRC, although their clinical benefit depends strongly on molecular context and biomarker-guided patient selection.

## 4 Protein kinases and SMKIs in CRC: biological rationale and therapeutic context

### 4.1 Protein kinases in CRC: roles in development and progression

Protein kinases are enzymes that transfer phosphate groups from adenosine triphosphate (ATP) to specific substrates, most commonly proteins. This phosphorylation process regulates biological functions such as cell proliferation, differentiation, metabolism, and apoptosis (Sarkar et al., 2023). Research shows that abnormal activation of kinase-driven signaling leads to malignant features such as excessive tumor cell growth, increased survival, angiogenesis, and promotion of metastasis. In CRC, hyperactivation of kinases associated with the mitogen-activated protein kinase (MAPK) pathway, the phosphatidylinositol 3-kinase/protein kinase B/mammalian target of the rapamycin (PI3K/AKT/mTOR, PAM) pathway, and the Wnt/β-catenin signaling cascade is particularly pronounced (Hayakawa et al., 2010; Narayanankutty, 2019; Liu et al., 2022). Since the beginning of the 21st century, kinase inhibitors developed to target protein kinases have emerged as an important class of potential anticancer agents and have been incorporated into the treatment of several malignancies (Attwood et al., 2021; Zhu et al., 2025). Among these, SMKIs are of potential relevance to CRC therapy owing to their oral availability, favorable pharmacokinetic properties, and precise modulation of key signaling networks.

### 4.2 Development and classification of SMKIs

**FIGURE 1:**
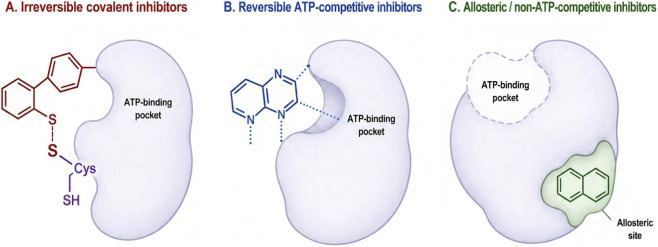
Schematic classification of SMKIs according to kinase-binding mode. **(A)** Irreversible covalent inhibitors form covalent bonds with specific amino acid residues, commonly cysteine (Cys), within or near the ATP-binding pocket. The solid connecting line indicates covalent attachment. **(B)** Reversible ATP-competitive inhibitors bind the ATP-binding pocket through non-covalent interactions. The dotted lines indicate reversible non-covalent interactions, such as hydrogen bonds, hydrophobic interactions, electrostatic forces, or van der Waals interactions. **(C)** Allosteric or non-ATP-competitive inhibitors bind regulatory sites outside the ATP-binding pocket and modulate kinase activity without directly competing with ATP. Abbreviations: SMKIs, small-molecule kinase inhibitors; Cys, cysteine.

Since the approval of the first BCR-ABL–targeting kinase inhibitor, imatinib, by the U.S. Food and Drug Administration (FDA) in 2001, SMKIs have evolved into a cornerstone of precision oncology (Cohen et al., 2021). As of 2023, more than 80 distinct SMKIs have received FDA approval worldwide. However, in the field of CRC, the number of FDA-approved SMKIs with explicit CRC indications remains limited, mainly in refractory metastatic colorectal cancer (mCRC) or rare biomarker-defined subgroups. SMKIs exert their effects either by competitively occupying the kinase ATP-binding site or by binding allosteric regulatory sites, thereby blocking abnormal signaling and regulating proliferation and apoptosis (Roskoski, 2024). SMKIs are generally small (< 500 Da), highly selective, orally administrable, and can sometimes cross the blood–brain barrier, features that have opened new prospects for precision therapy of central nervous system (CNS) tumors (Bedard et al., 2020). Depending on the mode of drug–kinase interaction, SMKIs can be broadly classified into reversible inhibitors, irreversible inhibitors, and allosteric/non-ATP-competitive inhibitors (Wu et al., 2015). Reversible inhibitors primarily interact with the kinase domain through noncovalent mechanisms, such as hydrogen bonds, hydrophobic interactions, electrostatic forces, and van der Waals interactions. Their efficacy is typically dependent on concentration and can be more easily modulated; however, they may be constrained by factors such as short target residence time, competition with ATP, mutations within the kinase domain, and compensatory signaling pathways (Attwood et al., 2021; Cohen et al., 2021). In contrast, irreversible inhibitors form covalent bonds with specific amino acid residues, most frequently cysteine residues, located within or adjacent to the kinase active site. This covalent interaction can lead to sustained target inhibition and extended pharmacodynamic effects, but it may also elevate the risk of off-target toxicity and the emergence of resistance due to mutations that interfere with covalent binding or activate alternative signaling pathways (Singh et al., 2011; Liu Q. et al., 2013). Allosteric or non-ATP-competitive inhibitors bind regulatory sites outside the ATP-binding pocket or stabilize inactive kinase conformations, which may improve selectivity but remains highly dependent on pathway context and resistance mechanisms. The structural features of representative drug–kinase interaction modes are illustrated in Figure 1, and their pharmacological advantages, limitations, representative agents, and CRC relevance are summarized in Table 1.

**TABLE 1:** Classification of small-molecule kinase inhibitors according to drug–kinase interaction mode.

Class	Binding mode	Pharmacological advantages	Major limitations	Representative drugs	Clinical relevance
Reversible ATP-competitive inhibitors	Bind ATP-binding pocket through noncovalent interactions	Controllable inhibition; dose adjustment feasible	ATP competition; short residence time; feedback activation	Regorafenib, fruquintinib, encorafenib	Used or investigated in selected CRC settings
Irreversible covalent inhibitors	Covalently bind cysteine or other residues near kinase active site	Sustained inhibition; longer pharmacodynamic effect	Off-target toxicity; resistance mutations	Afatinib, neratinib, pyrotinib	Mainly explored in EGFR/HER2-related CRC
Allosteric/ non-ATP-competitive inhibitors	Bind regulatory sites outside ATP pocket	Improved selectivity; avoids ATP competition	Context-dependent efficacy; adaptive resistance	Trametinib, binimetinib, MK-2206	Mainly investigational or combination-based in CRC

### 4.3 Clinical applications of SMKIs in CRC

In recent years, SMKIs have been widely implemented in the treatment of CRC and have achieved notable advances; the multikinase inhibitors regorafenib and fruquintinib were approved by the FDA in 2012 and 2023, respectively, for third-line treatment of advanced CRC (Roskoski, 2024). More notably, the phase III BEACON CRC trial demonstrated that the doublet regimen of encorafenib combined with cetuximab achieved an objective response rate (ORR) of 19.5% and significantly extended progression-free survival (PFS) to 9.3 months in patients with *BRAF V600E*-mutant metastatic CRC (Attwood et al., 2021). These clinical study data robustly substantiate the pivotal role of SMKIs in the management of advanced CRC.

## 5 SMKIs in CRC: mechanisms and clinical progress

**FIGURE 2:**
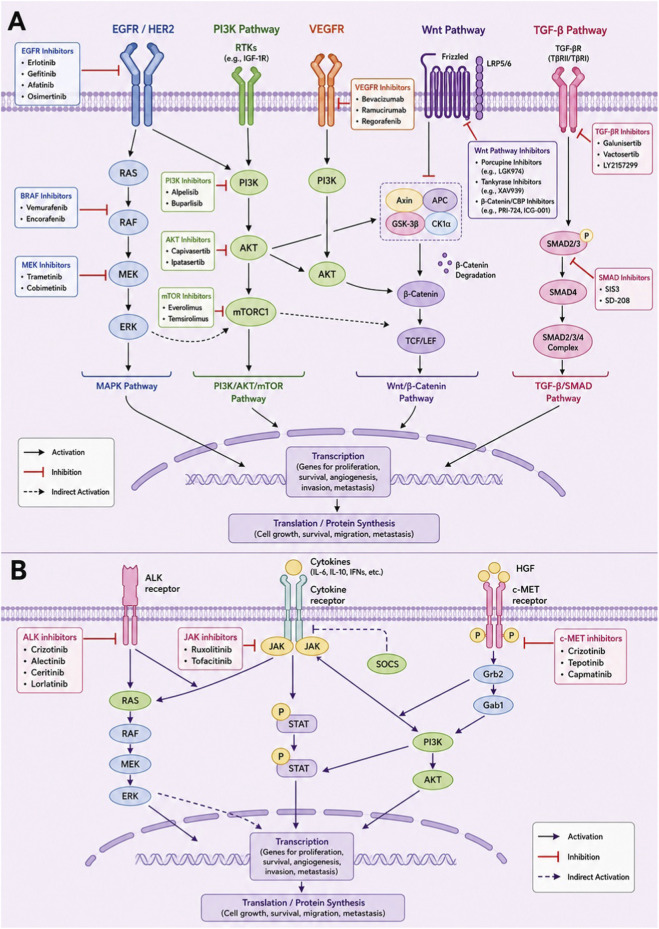
Integrated signaling pathways and representative small-molecule inhibitors in colorectal cancer. **(A)** Major CRC-related signaling pathways targeted by SMKIs, including EGFR/HER2-driven MAPK signaling, PI3K/AKT/mTOR signaling, VEGF/VEGFR-mediated angiogenic signaling, Wnt/β-catenin signaling, and TGF-β/SMAD signaling. These pathways regulate transcription, translation/protein synthesis, tumor-cell proliferation, survival, angiogenesis, invasion, and metastasis. Representative inhibitors are shown next to their corresponding targets, including EGFR inhibitors, BRAF inhibitors, MEK inhibitors, PI3K inhibitors, AKT inhibitors, mTOR inhibitors, VEGFR inhibitors, Wnt-pathway inhibitors, and TGF-β/SMAD pathway inhibitors. **(B)** Additional kinase-related pathways implicated in CRC progression and therapeutic resistance, including ALK-associated signaling, cytokine receptor/JAK/STAT signaling, SOCS-mediated feedback regulation, and HGF/c-MET-driven signaling. These pathways converge on MAPK, PI3K/AKT, and STAT-dependent transcriptional programs, thereby contributing to tumor growth, survival, angiogenesis, invasion, metastasis, and drug resistance. Solid arrows indicate pathway activation, red blunt-ended lines indicate pharmacological inhibition, and dashed arrows indicate indirect or compensatory activation. Abbreviations: CRC, colorectal cancer; SMKI, small-molecule kinase inhibitor; EGFR, epidermal growth factor receptor; HER2, human epidermal growth factor receptor 2; VEGFR, vascular endothelial growth factor receptor; MAPK, mitogen-activated protein kinase; PI3K, phosphoinositide 3-kinase; AKT, protein kinase B; mTOR, mammalian target of rapamycin; TGF-β, transforming growth factor-β; SMAD, suppressor of mothers against decapentaplegic; ALK, anaplastic lymphoma kinase; JAK, Janus kinase; STAT, signal transducer and activator of transcription; SOCS, suppressor of cytokine signaling; HGF, hepatocyte growth factor; c-MET, mesenchymal–epithelial transition factor.

This section is organized according to major signaling pathways and integrates mechanistic insights with clinical evidence. For each pathway, we first outline its biological role in CRC progression and resistance, followed by a discussion of the available clinical evidence for SMKIs-based interventions. Significantly, therapeutic success is not solely determined by pathway activation or target expression. In CRC, treatment response is influenced by molecular context, pathway redundancy, compensatory feedback activation, tumor microenvironment-mediated resistance, epigenetic regulation, drug-related toxicity, and tumor heterogeneity. Consequently, the mechanistic rationale serves as the groundwork for therapeutic advancements, with clinical benefits evaluated considering the available trial data and its limitations. To provide an integrated overview of the major signaling networks discussed in this section, we summarized the key CRC-related pathways and representative SMKIs in Figure 2.

### 5.1 Targeting core driver pathways in CRC: mechanisms and therapies

#### 5.1.1 MAPK signaling pathway

The MAPK signaling cascade plays a pivotal role in CRC progression (Samatar and Poulikakos, 2014). Epidermal growth factor receptor (EGFR) is overexpressed in approximately 65%–80% of patients with mCRC; however, only patients whose *RAS* and *BRAF* genes are wild-type derive benefit from anti-EGFR therapies (Ciardiello and Tortora, 2008). Clinical data show that *KRAS* mutations are present in approximately 40%–50% of CRC patients; these mutations lead to constitutive RAS activation that drives tumor growth independently of EGFR signaling and is associated with chemoresistance (Punekar et al., 2022). Additionally, approximately 8%–12% of patients harbor the *BRAF V600E* mutation—more commonly observed in right-sided colon cancers—which constitutively activates the MEK/ERK pathway as RAS-independent BRAF monomers and links to aggressive behavior, poor prognosis, and an immunosuppressive tumor microenvironment (Samatar and Poulikakos, 2014). Hence, even though MAPK signaling is fundamentally important to CRC biology, its therapeutic inhibition needs to be understood in the context of molecular specifics.

EGFR is a member of the erythroblastosis oncogene B (ERBB)/human epidermal growth factor receptor (HER) family (Arteaga and Engelman, 2014; Roskoski, 2019). Epidermal growth factor receptor tyrosine kinase inhibitors (EGFR-TKIs) have evolved through three generations. The first generation comprises reversible inhibitors—exemplified by gefitinib and erlotinib—which are active against EGFR wild-type and certain mutant forms but are prone to acquired resistance; The second generation comprises irreversible inhibitors—represented by afatinib and neratinib—that covalently bind the EGFR kinase domain and inhibit ERBB family members, exhibiting greater potency but increased toxicity. The third generation consists of T790M-selective inhibitors—including osimertinib, furmonertinib, and aumolertinib—which demonstrate notable efficacy against T790M-mediated resistance (Eide et al., 2020; Zhou et al., 2025). However, single-agent response rates to EGFR-TKIs in CRC are relatively low, which may be attributable to compensatory activation of receptor tyrosine kinases (RTKs) or feedback upregulation of MEK/ERK signaling (Kim et al., 2020). At present, clinical studies are mainly focused on combining EGFR TKIs with other therapies. Refer to Table 2 for specific regimens and data.

**TABLE 2:** Small-molecule protein inhibitors targeting the MAPK pathway in CRC.

SMKIs	NCT number	Targets	Phase	Subject (n)	Treatment	ORR	OS	PFS	Evidence level	Interpretation/Comment
Erlotinib	NCT03086538 (Kim et al., 2020)	EGFR	II	EGFR 3+ mCRC (n = 29)	Pemetrexed + Erlotinib	37.5%	7.7 m	2.6 m	Preliminary clinical signal	EGFR-expression selection produced an early response signal, but the small cohort and short PFS limit broad applicability.
Erlotinib	NCT00265824 (Tournigand et al., 2015)	EGFR	III	mCRC (n = 700)	Bevacizumab + Erlotinib	NA	24.9 m	5.4 m	Moderate clinical evidence	Large randomized evidence, but without clear biomarker enrichment; any incremental benefit may be diluted in molecularly unselected CRC.
Erlotinib	NCT00116506 (Meyerhardt et al., 2007)	EGFR	II	mCRC (n = 35)	FOLFOX + Bevacizumab + Erlotinib	34%	NA	NA	Exploratory	Small exploratory combination study; ORR cannot be confidently attributed to erlotinib because chemotherapy and anti-VEGF therapy contribute.
Erlotinib	NCT00940316	EGFR	II	CRC (n = 28)	Erlotinib + Panitumumab + Irinotecan	NA	12.6 m	4.6 m	Exploratory	Multi-agent regimen in a small cohort; toxicity, treatment backbone effects, and limited molecular selection restrict interpretation.
Erlotinib	NCT01135498 (Muñoz et al., 2013)	EGFR	II	mCRC (n = 35)	Bevacizumab + Erlotinib	NA	25.8 m	9.2 m	Preliminary clinical signal	Encouraging survival signal in a small study, but non-randomized/small-cohort evidence and lack of predictive biomarkers limit confidence.
Erlotinib	NCT00047762 (Hanauske et al., 2007)	EGFR	I	mCRC (n = 23)	FOLFOX + Erlotinib	NA	NA	NA	Exploratory	Dose/safety-oriented early-phase study; efficacy conclusions cannot be established.
Erlotinib	NCT00598156 (Johnsson et al., 2013)	EGFR	III	mCRC (n = 249)	Bevacizumab + Erlotinib	NA	26.7 m	5.7 m	Moderate clinical evidence	Randomized evidence exists, but weak molecular stratification leaves the incremental benefit over anti-VEGF-based therapy uncertain.
Afatinib	NCT01152437 (Hickish et al., 2014)	EGFR	II	wtKRAS mCRC (n = 50)	Afatinib	NA	11.7 m	1.5 m	Limited/ negative evidence	Low PFS despite KRAS selection suggests EGFR-TKI monotherapy is insufficient and constrained by bypass signaling.
Vemurafenib	NCT02164916 (Kopetz et al., 2021)	BRAF	II	BRAF V600E mCRC (n = 106)	Vemurafenib + Cetuximab + Irinotecan	17%	NA	4.2 m	Moderate clinical evidence	Biomarker-selected regimen with activity, but benefit remains modest, consistent with EGFR feedback and pathway redundancy.
Dabrafenib	NCT01750918 (Huijberts et al., 2020)	BRAF	I/II	BRAF V600E mCRC (n = 142)	Dabrafenib + Trametinib + Panitumumab	21%	9.1 m	4.2 m	Preliminary clinical signal	Vertical MAPK blockade shows activity, but early-phase design, toxicity, and adaptive resistance limit certainty.
Encorafenib	NCT04607421 (Kopetz et al., 2025)	BRAF	III	mCRC (n = 479)	Encorafenib + Cetuximab + mFOLFOX6	60.9%	10.3 m	NA	Relatively mature evidence	Large trial evidence indicates a strong signal when used in BRAF V600E molecularly selected disease; applicability depends on strict subgroup definition.
Encorafenib	NCT02928224 (Kopetz et al., 2019)	BRAF	III	BRAF V600E mCRC (n = 224)	Encorafenib + Binimetinib + Cetuximab	26%	9.0 m	4.3 m	Relatively mature evidence	Validated BRAF V600E strategy; benefit is clinically meaningful but not curative, and resistance still emerges.
Binimetinib	NCT03693170 (Van Cutsem et al., 2023)	MEK	II	BRAF V600E mCRC	Encorafenib + Binimetinib + Cetuximab	47.4%	18.3 m	5.8 m	Moderate clinical evidence	Biomarker-selected phase II activity is promising, but requires confirmation and careful comparison across trial settings.
Binimetinib	NCT02928224	MEK	III	BRAF V600E mCRC (n = 665)	Encorafenib + Cetuximab + Binimetinib	26.8%	9.3 m	4.3 m	Relatively mature evidence	Phase III evidence supports combined BRAF/EGFR with or without MEK inhibition, but modest PFS and toxicity highlight residual resistance.
Cobimetinib	NCT02788279	MEK	III	MSS mCRC (n = 183)	Atezolizumab + Cobimetinib	NA	8.87 m	1.9 m	Limited/ negative evidence	MSS immune-cold biology and lack of robust biomarker selection likely explain limited benefit of the MEK-ICI combination.
Selumetinib	NCT01116271 (Hochster et al., 2015)	MEK	I/II	KRAS mutated CRC (n = 31)	Selumetinib + Irinotecan	9.7%	NA	NA	Limited/ negative evidence	Low ORR in KRAS-mutant CRC reflects incomplete MAPK suppression and compensatory parallel signaling at tolerable doses.

NA, not available. Evidence level was qualitatively assigned according to trial phase, sample size, molecular selection, consistency of efficacy outcomes, and current clinical relevance, rather than formal GRADE-based evidence assessment. Categories: Exploratory = early-phase or immature evidence with limited validation; Preliminary clinical signal = small clinical studies showing potential activity but requiring confirmation; Moderate clinical evidence = reproducible efficacy signals from phase II, or biomarker-selected studies; Relatively mature evidence = larger trials, clinically meaningful outcomes, or partial practice integration; Limited/negative evidence = minimal efficacy, inconsistent outcomes, or studies limited by toxicity, resistance, or inadequate molecular selection.

Integrated interpretation: Overall, MAPK-directed SMKIs, in CRC, show highly context-dependent efficacy. EGFR-TKI-based regimens generally provide limited and inconsistent benefit, partly because many studies lack strict genomic selection and because downstream RAS/BRAF, activation; EGFR, feedback; PAM pathway compensation, and parallel RTK, signaling can rapidly restore proliferative signaling. More convincing activity has been observed in BRAF V600E-mutant CRC, treated with combined BRAF, EGFR, and/or MEK, inhibition; however, even these biomarker-selected strategies yield variable ORR, and often modest PFS/OS, gains. The limited durability of response likely reflects pathway reactivation, tumor heterogeneity, clonal evolution, overlapping toxicity, dose constraints, and differences in treatment line and trial design. Therefore, the clinical value of MAPK-targeted SMKIs, should be interpreted through molecular stratification and prospective validation rather than by pathway rationale alone.

BRAF is a serine/threonine kinase belonging to the RAF kinase family and plays a central role in the MAPK signaling pathway (Tosuner et al., 2018). BRAF inhibitors can be classified into two categories according to their target profiles: non-specific BRAF inhibitors and selective BRAF inhibitors. Non-specific BRAF inhibitors act by inhibiting multiple kinases, including BRAF, exhibiting broad antitumor and anti-angiogenic activities. They are used to treat various solid tumors such as hepatocellular carcinoma, renal cell carcinoma, thyroid cancer, and colorectal cancer. Selective BRAF inhibitors include vemurafenib, dabrafenib, and encorafenib. The *BRAF V600E* mutation occurs in 8%–15% of mCRC cases and commonly results in phosphorylation of mitogen-activated protein kinase (MEK) and extracellular signal-regulated kinase (ERK), provoking constitutive activation of the MAPK signaling pathway that drives tumor cell proliferation and survival and is associated with poor overall prognosis (Ros et al., 2021; Tabernero et al., 2021). However, the presence of *BRAF V600E* does not imply that BRAF inhibitor monotherapy is sufficient in CRC. Unlike melanoma, CRC rapidly develops EGFR-mediated MAPK feedback activation after BRAF blockade, thereby limiting the clinical efficacy of single-agent BRAF inhibition. Clinical trials for BRAF inhibitors in CRC are currently concentrating on combination treatments, notably BRAF and EGFR inhibition, with or without the addition of MEK inhibition. Current evidence indicates that these combinations may enhance results in certain patients with *BRAF V600E*-mutant mCRC, though the extent and persistence of the benefits differ among studies. Details of combination strategies incorporating BRAF inhibitors in CRC are summarized in Table 2.

MEK1/2 are dual-specificity kinases that mediate signal transduction within the MAPK pathway by phosphorylating ERK1/2 on the Thr-Glu-Tyr activation loop (Lian et al., 2019). Currently, four MEK inhibitors have been approved by the FDA for tumor-targeted therapy. Trametinib, Cobimetinib, Selumetinib, and Binimetinib are representative MEK1/2 inhibitors (Wang et al., 2021). However, in *RAS*-mutant CRC, MEK inhibitor monotherapy generally produces low response rates because of compensatory feedback activation and parallel pathway signaling (Wang et al., 2023). Therefore, the clinical use of MEK inhibitors in CRC has mainly focused on combination strategies with BRAF or EGFR inhibitors in molecularly selected settings. For example, in *BRAF V600E*-mutant metastatic CRC, combinations including encorafenib, cetuximab, and a MEK inhibitor have been reported to show antitumor activity (Van Cutsem et al., 2023). This combination strategy simultaneously targets multiple critical nodes within the MAPK pathway to block downstream signaling and thereby overcome the limitations of single-agent therapy.

In CRC, SMKIs targeting the MAPK pathway demonstrate significant clinical benefits predominantly in molecularly selected contexts, particularly in cases of *BRAF V600E*-mutant mCRC treated with a combination of BRAF and EGFR inhibitors. Conversely, EGFR-TKIs and MEK inhibitors have shown limited or inconsistent efficacy in unselected patient populations. The observed low response rates and marginal survival benefits may be attributed to downstream *RAS/BRAF* mutations, compensatory activation of the PAM pathway, inadequate molecular stratification, and dose modifications due to toxicity in multi-agent treatment regimens.

#### 5.1.2 PAM signaling pathway

The PAM signaling pathway maintains cellular homeostasis by regulating key processes, including metabolism, proliferation, survival, and resistance to apoptosis. Ligand-induced RTK autophosphorylation recruits PI3K (composed of a regulatory p85 subunit and a catalytic p110 subunit) via the adaptor proteins Grb2, catalyzing the conversion of PIP2 to the second messenger PIP3 (Burke, 2018), PIP3 engages the AKT PH domain, facilitating AKT activation by PDK1 and mTORC2; AKT then phosphorylates TSC1/2 to activate mTORC1 (Hemmings and Restuccia, 2015; Yu et al., 2022). Notably, *PTEN* is a critical negative regulator of this pathway, acting by terminating signal transduction by catalyzing the dephosphorylation of PIP3 to generate PIP2 (Chen et al., 2019). Studies have demonstrated that dysregulation of the PAM pathway—such as *PTEN* loss, *PIK3CA* mutations, AKT amplification, or TSC1/2 loss—leads to sustained pathway activation that drives progression of various malignancies (Nunnery and Mayer, 2020; Tewari et al., 2022; Hashemi et al., 2023; Leiphrakpam and Are, 2024). In CRC, approximately 15%–20% of patients harbor *PIK3CA* exon 9 or 20 mutations, and about 30% exhibit loss of *PTEN* expression or functional inactivation, cumulatively resulting in pronounced activation of the PAM pathway (Glaviano et al., 2023). Pathway-driven signal amplification not only directly promotes proliferative and survival programs but also induces widespread metabolic reprogramming, with fatty-acid metabolism being particularly critical: tumors upregulate *de novo* lipid biosynthesis (the SREBP–FASN axis), increase fatty-acid uptake and *β*-oxidation, and remodel membrane phospholipid composition and lipid-raft architecture, thereby providing substrates for membrane biogenesis, energy production, and one-carbon metabolism while stabilizing RTK clustering in the membrane to reinforce RTK–PI3K complex formation and sustain AKT/mTOR signaling (Koundouros and Poulogiannis, 2020). Moreover, lipid metabolites can shape an immunosuppressive tumor microenvironment that indirectly supports persistent activation of the signaling axis—the aforementioned metabolic compensation forms a metabolic resistance barrier to PAM axis inhibition (Cuyàs et al., 2024). Based on their molecular targets, PAM-pathway inhibitors are classified according to target specificity into PI3Kα-selective inhibitors, AKT allosteric inhibitors, and mTORC1 inhibitors (Table 3).

**TABLE 3:** Small molecule protein inhibitors targeting the PAM pathway in CRC.

SMKIs	NCT number	Targets	Phase	Subject (n)	Treatment	ORR	OS	PFS	Evidence level	Interpretation/Comment
Alpelisib	NCT01719380 (van Geel et al., 2017)	PI3Kα	Ib	BRAF V600E mCRC (n = 26)	Encorafenib + Cetuximab + Alpelisib	18%	NA	4.2 m	Preliminary clinical signal	Early-phase, biomarker-selected triplet study showing modest activity; the incremental contribution of PI3Kα inhibition remains uncertain because of small sample size and combination design.
Everolimus	NCT01139138 (Townsend et al., 2018)	mTOR	Ib/II	mCRC	Panitumumab + Irinotecan + Everolimus	NA	11.8 m	6.4 m	Exploratory	Combination showed a disease-control signal, but no clear independent incremental benefit of everolimus can be inferred without stronger comparative evidence and biomarker selection.
Everolimus	NCT00419159	mTOR	II	mCRC (n = 99)	Everolimus	NA	4.9 m	1.8 m	Limited/ negative evidence	Single-agent mTOR inhibition produced limited clinical benefit, consistent with feedback reactivation and incomplete pathway suppression at tolerable doses.
Temsirolimus	NCT00827684 (Spindler et al., 2013)	mTOR	II	KRAS mutant mCRC (n = 99)	Temsirolimus + Irinotecan	NA	5.3 m	NA	Limited/ negative evidence	Despite KRAS-mutant enrichment, available outcomes do not support a robust benefit; interpretation is limited by absent ORR/PFS reporting and lack of validated predictive biomarkers.
MK-2206	NCT01333475 (Do et al., 2015)	AKT	II	mCRC (n = 21)	MK-2206+Selumetinib	NA	NA	1.9 m	Limited/ negative evidence	Dual AKT/MEK inhibition showed poor activity in a small study, highlighting tolerability, pathway redundancy, and insufficient molecular selection as major barriers.

Integrated interpretation. Overall, PAM-targeted SMKIs, have shown limited and inconsistent efficacy in CRC, despite a strong mechanistic rationale. Low ORR, and short PFS, may reflect pathway redundancy; MAPK, feedback activation, incomplete PI3K/AKT/mTOR, blockade at tolerable doses, metabolic compensation, and heterogeneity of PIK3CA/PTEN, alterations. Many studies were early-phase, small, or only partially biomarker-selected, which may dilute potential benefit in truly sensitive subgroups. Future PAM-pathway strategies require stricter molecular selection, toxicity-aware dosing, and rational combinations validated prospectively.

PI3Kα-selective inhibitors (e.g., Alpelisib, Buparisib, and Copanlisib) suppress PI3Kα activity by targeting *PIK3CA* mutations, thereby blocking downstream AKT/mTOR signal transduction. Although these agents have been approved for *PIK3CA*-mutant patients in breast cancer, studies in CRC demonstrate very limited single-agent efficacy, which may be related to tumor metabolic characteristics (André et al., 2019). Studies have confirmed that PI3Kα inhibition induces a treatment-related hyperglycemia–insulin feedback loop. In *PTEN*-deficient CRC, increased insulin reactivates the INSR/IGF1R–PI3K/AKT axis, thereby counteracting the effects of PI3Kα inhibitors; dietary or pharmacologic control of blood glucose and insulin can restore tumor sensitivity to PI3Kα inhibition, providing a viable rationale for combined metabolic-management strategies in CRC (Hopkins et al., 2018). Buparisib is an oral, reversible pan-class I PI3K inhibitor; preclinical studies demonstrate antiproliferative activity in *PIK3CA*-mutant cancer cell lines, but its clinical utility in CRC requires validation in large-scale clinical datasets (Liu N. et al., 2013). PI3Kα inhibition may attenuate efficacy via compensatory activation of the MAPK pathway. Consequently, combination with MEK inhibitors to suppress ERK signaling is proposed to enhance antitumor activity (Juric et al., 2018; Whitehead et al., 2024), but this approach remains investigational in CRC and requires further clinical validation.

Dual PI3K/mTOR inhibitors (e.g., Dactolisib, Gedatolisib, and Samotolisib) simultaneously target PI3K catalytic isoforms (p110) and mTORC1/2 complexes (Wu et al., 2022). These inhibitors are currently in clinical trials; however, multiple preclinical and early clinical studies have reported poor tolerability and/or limited antitumor activity in patients with advanced or metastatic solid tumors (Wainberg et al., 2017; Azaro et al., 2021).

AKT inhibitors concentrate on targeting AKT (also known as PKB or Rac), a protein kinase that mediates cellular functions including proliferation, survival, and metabolism (Shariati and Meric-Bernstam, 2019). Small-molecule AKT inhibitors are classified into allosteric inhibitors (e.g., Perifosine, Ipatasertib) and ATP-competitive inhibitors (e.g., MK-2206, Ladirsertib) (Lazaro et al., 2020). Ipatasertib is an AKT allosteric inhibitor that suppresses AKT activity via an allosteric mechanism, thereby blocking downstream mTORC1 signaling (Sun L. et al., 2018). Although AKT inhibitors have been employed as monotherapies in gastric, breast, and prostate cancers, their application in CRC remains at the clinical-trial stage (Hossain, 2024). In PTEN-deficient CRC, loss of *PTEN* function leads to hyperactivation of the PI3K/AKT pathway. Studies indicate that combining Ipatasertib with chemotherapy (e.g., mFOLFOX6) can significantly prolong PFS (Lazaro et al., 2020). AKT inhibitors exert synergistic effects by inhibiting tumor cell proliferation and angiogenesis, and they display high selectivity for *PTEN*-deficient tumors. However, their efficacy may be influenced by tumor heterogeneity and resistance mechanisms, necessitating guidance from genomic profiling for patient selection.

The mTOR inhibitors (Saxton and Sabatini, 2017) target the key downstream regulator mTOR (the mTORC1 and mTORC2 complexes). Mechanistically, mTORC1 regulates translational elongation via the S6K-eEF2B axis, and adenomatous polyposis coli (APC)-deficient CRC cells depend on this pathway to sustain proliferation (Holz et al., 2021). Specifically, mTOR inhibitors include rapamycin, everolimus, temsirolimus, and vistusertib, among others. Rapamycin inhibits mTORC1 activity, blocks eEF2 phosphorylation, resulting in tumor growth arrest and promotion of differentiation (Faller et al., 2015). In addition, mTORC1 promotes tumor proliferation by activating ribosome biogenesis, and targeting this pathway can inhibit colorectal cancer cell growth (Wang et al., 2020). Rapamycin caused a halt in tumor cell growth and regression in APC-deficient mice with intestinal adenomas, and inhibiting mTORC1 increased survival in mouse models treated with chemotherapy (Faller et al., 2015; Schmitt et al., 2022). However, these mechanistic and preclinical findings have not translated into broad clinical success in CRC. The effectiveness of rapamycin and its derivatives in certain CRC patient populations is limited or preliminary, showing only modest results as single agents, which calls for improved combination strategies (Naing et al., 2012). Everolimus may induce compensatory MAPK pathway activation after mTORC1 inhibition, further illustrating feedback complexity (Carracedo et al., 2008). Currently, everolimus is mainly used as a second-line treatment for advanced or refractory CRC (Weldon Gilcrease et al., 2019). An IB/II study in KRAS-wildtype mCRC reported (Townsend et al., 2018) that the combination of panitumumab (an anti-EGFR monoclonal antibody), irinotecan, and everolimus achieved a response rate (RR) of 60%, an OS of 11.8 months, and a PFS of 6.4 months. However, these outcomes were comparable to those of irinotecan plus anti-EGFR therapy, indicating no clearly proven incremental benefit from adding everolimus. Everolimus combined with chemotherapy has shown only preliminary disease-control activity, and its clinical application remains limited by early-phase evidence, dosing optimization, toxicity concerns, and lack of validated biomarkers (Fritsch et al., 2023).

Although dysregulation of the PAM signaling pathway is common in CRC, SMKIs targeting this pathway have not consistently yielded clinical benefits. Tumors harboring *PIK3CA* mutations, *PTEN* loss, AKT activation, or mTOR dependency may represent potentially responsive subgroups; however, these biomarkers have not been sufficiently validated for routine clinical selection. The limited efficacy of PAM-directed SMKIs may be due to MAPK pathway feedback activation, metabolic compensation, reactivation mediated by IGF1R, pathway redundancy, and dose-limiting toxicities such as hyperglycemia, rash, diarrhea, and fatigue.

#### 5.1.3 VEGF/VEGFR pathway

The vascular endothelial growth factor (VEGF)/VEGF receptors (VEGFR) pathway constitutes the central regulatory mechanism of tumor angiogenesis. Upon binding of VEGF to VEGFR-2, downstream signaling cascades such as PAM and MAPK are activated, which promote endothelial cell proliferation, migration, and alterations in vascular permeability, thereby supplying tumors with nutrients and facilitating metastatic dissemination (Ferrara et al., 2003). Furthermore, this pathway contributes to tumor immune evasion by modulating the tumor immune microenvironment—for example, by inhibiting T-cell infiltration (Voron et al., 2015). In CRC, VEGF is overexpressed in the tumor microenvironment in approximately 60% of patients, a finding that is significantly associated with an increased risk of liver metastasis (Cascinu et al., 2000). Hypoxia and inflammatory mediators induce VEGF secretion, forming a “angiogenesis-metastasis” vicious cycle (Zhang Z. et al., 2025). VEGFR-2 plays a central role in angiogenesis in CRC; its phosphorylation activates downstream signaling cascades that drive pathological tumor neovascularization.

Inhibitors of the VEGF/VEGFR pathway principally block signaling by two mechanisms. One class comprises monoclonal antibodies such as bevacizumab (a monoclonal antibody targeting VEGF-A), aflibercept (a recombinant decoy receptor that binds VEGF-A/B and placental growth factor, PlGF), and ramucirumab (a monoclonal antibody specific for VEGFR-2); these agents suppress angiogenesis by neutralizing ligands or preventing ligand–receptor interactions. The other class consists of small-molecule TKIs, including regorafenib and fruquintinib; these agents competitively bind the tyrosine-kinase domain and thereby prevent activation of downstream signaling cascades. Mechanistically, VEGF-A inhibitors may potentiate the efficacy of PD-1/PD-L1 antibodies by normalizing the tumor vasculature, increasing T-cell infiltration, and reducing the activity of immunosuppressive cell populations (Kim et al., 2019).

Currently, multi-target VEGFR inhibitors impede angiogenesis by inhibiting VEGFR1–3, FGFRs, and other kinases, and have been approved for later-line treatment of advanced CRC. The phase III FRESCO trial demonstrated the efficacy of fruquintinib, which has been approved in China for third-line or later treatment of mCRC (Shirley, 2018). The phase-III CORRECT trial likewise demonstrated that regorafenib can prolong survival in patients with disease progression after standard chemotherapy (Shin et al., 2023). However, the overall benefit is typically small, and objective response rates are low, implying that these agents mainly aid in maintaining disease stability instead of causing significant tumor reduction (Li et al., 2018). Compared with antibody-based anti-VEGF therapies, VEGFR-targeted SMKIs offer oral administration and broader kinase coverage, but they are also associated with class-specific toxicities and limited response rates. See Table 4 for detailed applications of specific agents in CRC.

**TABLE 4:** Small molecule protein inhibitors targeting the VEGF pathway undergo clinical studies in CRC.

SMKIs	NCT number	Targets	Phase	Subject (n)	Treatment	ORR	OS	PFS	Evidence level	Interpretation/Comment
Bevacizumab	NCT00109070 (Hurwitz et al., 2004)	VEGFR-A	III	mCRC (n = 402)	Bevacizumab + Irinotecan	44.8%	20.3 m	10.6 m	Relatively mature evidence	Randomized phase III evidence supports anti-VEGF pathway benefit; it should be interpreted as an anti-angiogenic comparator.
Lenvatinib	NCT04776148 (Kawazoe et al., 2024)	VEGFR	III	pMMR/ MSI-H mCRC (n = 480)	Ienvatinib + Pembrolizumab	10.4%	9.8 m	3.8 m	Limited/ negative evidence	Large phase III design, but modest ORR/PFS indicates limited broad benefit in unselected or insufficiently enriched mCRC.
Aflibercept	NCT00561470 (Van Cutsem et al., 2012)	VEGFR	III	mCRC (n = 612)	Aflibercept + FOLFIRI	19.8%	13.5 m	6.9 m	Relatively mature evidence	Phase III evidence supports modest survival benefit after prior therapy. Benefit is generally incremental.
Ramucirumab	NCT01183780 (Tabernero et al., 2015)	VEGFR-2	III	mCRC (n = 536)	Ramucirumab + FOLFIRI	13.4%	13.3 m	5.7 m	Relatively mature evidence	Randomized phase III evidence supports anti-VEGFR-2 pathway activity, but ramucirumab is an antibody; clinical benefit is survival-oriented rather than strongly cytoreductive.
Ramucirumab	NCT01103323	VEGFR-2	III	mCRC (n = 505)	Ramucirumab	1.0%	1.9 m	6.4 m	Limited/ negative evidence	Single-agent VEGFR-2 blockade showed very low ORR, suggesting limited cytoreductive activity and the need for careful source verification and combination context.
Regorafenib	NCT03406871 (Fukuoka et al., 2020)	VEGFR	Ib/II	MSS mCRC (n = 25)	Regorafenib + Nivolumab	36%	NA	7.9 m	Preliminary clinical signal	Small exploratory immunotherapy-TKI combination showed an activity signal in MSS mCRC, but results require validation in larger prospective studies.
Fruquintinib	NCT02314819	VEGFR	III	mCRC (n = 278)	Fruquintinib	4.7%	9.3 m	3.7 m	Relatively mature evidence	Phase III evidence supports later-line VEGFR TKI use; low ORR indicates mainly cytostatic activity with modest survival benefit rather than frequent tumor shrinkage.
Fruquintinib	NCT03903705 (Guo et al., 2023)	VEGFR	Ib/II	MSS mCRC (n = 44)	Fruquintinib + Sintilimab	20.0%	20.0 m	6.9 m	Preliminary clinical signal	Early-phase combination data suggest potential immune-angiogenic synergy, but the small sample size and non-randomized design limit generalizability.

Integrated interpretation. Across VEGF/VEGFR-directed studies, the evidence is strongest for anti-angiogenic pathway inhibition, but the magnitude of benefit is often modest and frequently cytostatic. Low ORR, with VEGFR TKIs, may reflect vascular normalization or disease stabilization rather than direct tumor-cell killing. OS/PFS, gains are influenced by line of therapy, chemotherapy backbone, performance status, and absence of reliable predictive biomarkers.

### 5.2 Applications of microenvironment- and stemness-related pathways in CRC

#### 5.2.1 Wnt/β-catenin pathway

The Wnt/β-catenin signaling pathway is a central regulator of stem cell self-renewal, cell polarity, and tissue homeostasis, and its aberrant activation is closely associated with CRC development (Liu et al., 2022). Mechanistically, upon Wnt engagement of Frizzled/LRP5/6, Dishevelled (DVL) is recruited, and the APC/axis inhibition protein (AXIN)/glycogen synthase kinase 3 beta (GSK3β) destruction complex is inhibited, preventing β-catenin phosphorylation and degradation. Stabilized β-catenin accumulates and translocates to the nucleus. There it binds T-cell factor (TCF)/lymphoid enhancer-binding factor (LEF) to drive targets such as MYC proto-oncogene (c-MYC) and Cyclin D1 (Song et al., 2024). In CRC, approximately 80% of sporadic cases harbor inactivating mutations in APC, which drive tumor stemness and proliferative behavior (Zhao H. et al., 2022). Additionally, roughly 5% of patients harbor activating mutations in *CTNNB1*, which directly stabilize β-catenin and promote tumor progression (Liang et al., 2019).

Strategies targeting the Wnt pathway operate on multiple levels: at the level of transcriptional regulation, aberrant Wnt/β-catenin activation is closely associated with CRC liver metastasis and chemoresistance, and Adavivint—by inhibiting the adavivint inhibits the a disintegrin and metalloproteinase domain-containing protein 10 (ADAM10)/notch receptor 2 (NOTCH2)/transcription factor 7-like 2 (TCF7L2) signaling axis—blocks transcription of Wnt target genes such as MYC, JUN and CCND1/2 by a mechanism independent of canonical β-catenin signaling, demonstrating both tumor-growth inhibition and chemosensitizing effects in CRC organoid models (Xiang et al., 2025). At the level of direct regulation of β-catenin stability, NU2058 targets RanBP3 to promote nuclear export of β-catenin, thereby suppressing c-Myc and cyclin D1 expression and inducing cellular senescence (Zheng et al., 2022), whereas compounds such as C644-0303 inhibit β-catenin phosphorylation and nuclear accumulation, downregulate target genes including Axin2 and Cyclin D1, arrest the cell cycle, and induce apoptosis (Yan et al., 2021). In terms of bypass regulation, crosstalk between the Wnt pathway and other signaling axes such as Notch and Hedgehog—for example, ADAM10/NOTCH2 signaling indirectly regulating Wnt targets via TCF7L2—offers novel strategies to overcome resistance; additionally, combining Wnt pathway inhibitors with MAPK pathway inhibitors may enhance therapeutic efficacy and reduce the emergence of resistance (Moon et al., 2014; Choi et al., 2020).

Although no SMKIs that directly target the Wnt/β-catenin pathway have yet received clinical approval, several Wnt-related small molecules have entered clinical or preclinical evaluation. Among the small molecules linked to Wnt/β-catenin, napabucasin is one of the few to progress to phase III clinical testing. It is not a conventional single-target inhibitor, but rather induces an NQO1-mediated burst of reactive oxygen species that leads to inactivation of both STAT3 and β-catenin signaling, thereby selectively killing NQO1-positive, cancer stem-cell (CSC)–enriched colorectal cancer cells and acting synergistically with radiochemotherapy or FOLFIRI. However, two phase-III studies, one evaluating napabucasin as monotherapy and another in combination with FOLFIRI, failed to demonstrate benefits in OS or objective response (Table 5). These negative results may reflect heavily pretreated populations, tumor heterogeneity, insufficient biomarker enrichment, pathway redundancy, and the limited ability of STAT3/β-catenin-associated inhibition to overcome multiple resistance mechanisms. Therefore, the mechanistic relevance of Wnt/β-catenin- and STAT3-associated signaling in CRC stemness and resistance does not necessarily translate into clinical efficacy without more precise biomarker selection and prospective validation. In addition, Porcupine inhibitors (e.g., LGK974, ETC-159) block Wnt signaling activation in *APC*-mutant CRC by inhibiting palmitoylation and secretion of Wnt ligands, and they have demonstrated significant tumor growth suppression in preclinical models (Park and Kim, 2023); β-catenin/TCF inhibitors (e.g., Tegavivint) disrupt the transcriptional complex formed by β-catenin and TCF/LEF, suppress downstream target gene expression, and are currently in early clinical development with investigations (Song et al., 2024). These therapeutic strategies offer new avenues for targeted treatment of CRC, but their clinical translation requires careful balancing of antitumor efficacy against potential adverse effects, including disruption of normal tissue homeostasis.

**TABLE 5:** Small molecule protein inhibitors targeting the microenvironment- and stemness-related pathways undergoing clinical studies in CRC.

SMKIs	NCT number	Targets	Phase	Subject (n)	Treatment	ORR	OS	PFS	Evidence level	Interpretation/Comment
Napabucasin	NCT01830621 (Jonker et al., 2018)	STAT3/β-catenin	III	mCRC (n = 144)	Napabucasin	1%	4.4 m	1.7 m	Limited/ negative evidence	Randomized phase III trial stopped for futility. Napabucasin did not improve OS in the unselected population.
Napabucasin	NCT02753127 (Shah et al., 2023)	STAT3/β-catenin	III	mCRC (n = 624)	Napabucasin + FOLFIRI	NR	11.4 m	NR	Limited/ negative evidence	Large randomized phase III CanStem303C study did not meet its primary OS endpoint in either the overall population or the pSTAT3-positive subgroup.
Ruxolitinib	NCT02119676 (David et al., 2018)	JAK	II	mCRC (n = 221)	Ruxolitinib + Regorafenib	2.7%	11.4 m	NA	Limited/ negative evidence	Prospective phase II study showed very low ORR, suggesting.
Vactosertib	NCT03724851 (Guven et al., 2024)	TGFβI	Ib/II	MSS mCRC (n = 33)	Vactosertib + Pembrolizumab	15.2%	15.8 m	1.3 m	Preliminary clinical signal	Early-phase MSS-enriched study suggests potential TGF-β/PD-1 synergy, but short PFS and small sample size require cautious interpretation and prospective validation.

Integrated interpretation. For microenvironment- and stemness-related pathways, most evidence remains negative, limited, or exploratory. Although STAT3/β-catenin, JAK/STAT, and TGF-β, signaling are biologically relevant to stemness, immune escape, stromal remodeling, and resistance, clinical translation has been constrained by pathway complexity, lack of validated predictive biomarkers, MSS, immune-cold biology, and heterogeneous patient selection.

#### 5.2.2 JAK/STAT pathway

The Janus kinase/signal transducer and activator of transcription (JAK/STAT signaling pathway) is principally associated with sustained activation of STAT3 and STAT5; these factors promote tumor progression by regulating proliferation (e.g., c-Myc, BCL2), inhibiting apoptosis (e.g., caspase family members), and inducing immune evasion (e.g., Treg differentiation, PD-L1 upregulation) (Hu X. et al., 2021). The JAK/STAT axis mediates signaling of inflammatory cytokines such as interleukin-6 (IL-6); activation of STAT3 via JAK1/JAK2 promotes an immunosuppressive tumor microenvironment (Xue et al., 2023). In CRC, elevated IL-6 levels are associated with persistent STAT3 phosphorylation; sustained STAT3 activation can induce PD-L1 expression, leading to T-cell exhaustion and suppression of antitumor immunity (Whitehead et al., 2024).

JAK/STAT pathway inhibitors are classifiable into two generations based on selectivity: first-generation non-selective inhibitors broadly inhibit JAK1/3 or JAK1/2 isoforms to block multiple signaling cascades, are principally used to treat ulcerative colitis, and are associated with notable adverse effects, such as an increased risk of infection (Sandborn et al., 2017). Among second-generation selective inhibitors, JAK1/2 inhibitors such as ruxolitinib specifically target signaling pathways implicated in myeloproliferative neoplasms (Mesa et al., 2017). Target tyrosine kinase 2 (TYK2) inhibitors such as deucravacitinib are mainly applied in immune-mediated diseases (Strober et al., 2023), whereas STAT3 inhibitors directly block STAT3 phosphorylation to abrogate its oncogenic functions but have limited *in vivo* utility due to pharmacokinetic constraints. Moreover, following JAK/STAT activation, suppressors of cytokine signaling (SOCS) proteins competitively bind phosphorylated JAK kinases or receptors to block further pathway activation, this mechanism modulates immune responses in CRC and can enhance chemosensitivity (Wang et al., 2024; Pandey et al., 2023), yet agents targeting SOCS proteins remain at the preclinical stage and warrant further investigation.

In CRC therapy, inhibitors of the JAK/STAT pathway are largely in preclinical development; for example, the natural compound baicalein suppresses the JAK2/STAT3 axis and downregulates the ferroptosis regulator glutathione peroxidase 4 (GPX4), significantly inhibiting tumor growth in animal models, although large-scale clinical validation is lacking (Lai et al., 2024). Ruxolitinib, also known as INCB018424, competitively binds the ATP-binding site of JAK kinases to block their phosphorylation activity, thereby inhibiting JAK/STAT signaling and interrupting intracellular signaling downstream of pro-inflammatory cytokines such as IL-6 and IFN-γ (Li et al., 2021). Current clinical exploration focuses on combination regimens; ruxolitinib combined with anti-PD-1 antibodies has shown preliminary synergistic effects in MSS CRC by reversing immunosuppression (Giordano et al., 2019). However, the available evidence remains preliminary, and JAK/STAT pathway inhibitors have not yet been established as a clinically validated SMKI-based therapeutic strategy for CRC.

#### 5.2.3 TGF-β pathway

TGF-β is a cytokine closely associated with EMT. The TGF-β pathway exerts tumor-suppressive effects in early stages via Smad2/3 phosphorylation to inhibit cell proliferation and maintain tissue homeostasis, whereas in advanced stages it promotes tumor cell invasion and metastasis by activating Smad4-dependent epithelial–EMT. In advanced CRC, the TGF-β pathway drives tumor progression via a dual mechanism: on one hand, it suppresses cytotoxic T-cell and natural killer (NK) cell activity while inducing expansion of regulatory T cells (Tregs) and polarization of tumor-associated macrophages toward an M2 phenotype, collectively establishing an immunosuppressive microenvironment; on the other hand, it activates cancer-associated fibroblasts (CAFs), promoting extracellular-matrix remodeling and fibrosis that form a physical barrier conducive to metastasis. These cooperative effects ultimately furnish tumor cells with both immune-evasive support and stromal structural backing for metastatic dissemination (Massagué and Sheppard, 2023). Furthermore, dysregulated TGF-β signaling enhances Cancer stem cell (CSC) properties by upregulating CD44 and sex-determining region Y-box 2 (SOX2) (Zhang et al., 2021).

TGF-β pathway inhibitors can be classified into four principal categories according to their molecular targets: (1) ligand/receptor binding agents, including neutralizing antibodies such as fresolimumab (GC1008) that block ligand–receptor interactions (Morris et al., 2014), and bifunctional fusion proteins such as M7824 (bintrafusp alfa), which sequesters TGF-β ligands via the extracellular domain of TGF-βRII while simultaneously blocking the PD-L1 axis (Lan et al., 2018). (2) Receptor kinase inhibitors, exemplified by galunisertib (LY2157299) and vactosertib, which selectively inhibit TGF-βRI (ALK5) and prevent SMAD2/3 phosphorylation (Yingling et al., 2017), while agents such as LY3200882 inhibit both TGF-βRI and TGF-βRII (Kim et al., 2021). (3) SMAD pathway modulators, including genetic regulators such as SMAD7, which competitively interferes with receptor-mediated R-SMAD phosphorylation, and small molecules such as SIS3, a selective inhibitor of SMAD3 phosphorylation (Jinnin et al., 2006). (4) Non-canonical pathway inhibitors, which indirectly attenuate TGF-β signaling by targeting parallel oncogenic cascades. Collectively, these diverse strategies highlight the multidimensional therapeutic potential of TGF-β pathway blockade in oncology.

In the context of CRC therapy, SMKIs of the TGF-β pathway have demonstrated heterogeneous clinical activity. Vactosertib, a selective TβRI inhibitor that competitively occupies the receptor ATP pocket to block downstream Smad2/3 phosphorylation, produced an ORR of 15.2% when combined with a PD-1 inhibitor in patients with MSS CRC (Guven et al., 2024). Moreover, Vactosertib (Binabaj et al., 2023) has been reported to enhance the antitumor activity of 5-fluorouracil (5-FU) against CRC cells by upregulating E-cadherin expression and inhibiting matrix metalloproteinase-9 (MMP-9) enzymatic activity. The TβRI inhibitor LY364947 has demonstrated anti-metastatic effects in preclinical models, although it has not yet advanced to phase-III clinical evaluation (Yang et al., 2022). Therefore, TGF-β pathway blockade is mechanistically promising in CRC, but it remains investigational and requires further clinical validation before it can be considered an established SMKIs-based therapeutic strategy.

### 5.3 Emerging molecular targets in CRC

#### 5.3.1 HER2

Human epidermal growth factor receptor 2 (HER2/ERBB2) is an oncogene encoding a transmembrane glycoprotein receptor with intrinsic tyrosine-kinase activity. HER2 primarily signals through MAPK, PAM, and JAK/STAT3 cascades to regulate cell proliferation, survival, and differentiation (Wang N. et al., 2022). In settings of HER2 gene amplification or protein overexpression, HER2 forms homo- or heterodimers (e.g., HER2–EGFR or HER2–HER3) that constitutively activate downstream signaling, promoting tumor cell proliferation and resistance to apoptosis and contributing to the pathogenesis of diverse malignancies (Chen et al., 2024). HER2 amplification has emerged as an important therapeutic target in CRC. The prevalence of HER2 amplification in CRC is approximately 1.3%–6%; HER2 overexpression correlates with increased tumor invasiveness, poorer prognosis, and an association with resistance to EGFR inhibitors (Zhang et al., 2024).

HER2-targeting SKMIs are categorized by selectivity and mechanism into three principal classes: (1) reversible dual-tyrosine-kinase inhibitors such as lapatinib, which target HER2 and EGFR simultaneously but have modest single-agent activity (Roskoski, 2014); (2) irreversible pan-ERBB inhibitors such as neratinib and pyrotinib that covalently inhibit HER1/HER2/HER4 kinase domains to prevent autophosphorylation and downstream signaling (Tiwari et al., 2016); and (3) highly selective HER2 inhibitors such as Tucatinib (Lee, 2020), which minimally inhibit EGFR and therefore exhibit reduced gastrointestinal and dermatologic toxicity relative to dual-target or pan-ERBB agents.

Targeted therapies for HER2-positive CRC have advanced substantially, particularly dual-target combination regimens (see Table 6), which more effectively suppress HER2 signaling and thereby enhance therapeutic efficacy. In the HERACLES trial, the combination of trastuzumab (an anti-HER2 monoclonal antibody) plus lapatinib produced an ORR of 28% and a PFS of 4.7 months (Sartore-Bianchi et al., 2016); similarly, the MOUNTAINEER study (NCT03043313) reported an ORR of 38.1% for trastuzumab combined with tucatinib (Strickler et al., 2023). The antibody-drug conjugate (ADC) trastuzumab deruxtecan (DS-8201)—already approved in breast cancer—has shown durable activity in the DESTINY-CRC01 trial for HER2-positive CRC patients refractory to standard therapies, achieving an ORR of 45.3% (Yoshino et al., 2023). However, these findings should not be generalized to unselected CRC populations. HER2 positivity is uncommon and biologically heterogeneous, and resistance may arise from inaccurate or variable biomarker selection, intratumoral HER2 heterogeneity, coexisting gene alterations, downstream MAPK or PAM pathway reactivation, bypass receptor tyrosine kinase activation, or loss of HER2 dependence during clonal evolution. In addition, emerging approaches—including combinations of immunotherapy with HER2-targeted agents and HER2-directed CAR-T therapies—are under active investigation. Thus, HER2 is a clinically actionable but uncommon target in CRC, and HER2-directed SMKIs strategies require precise molecular selection and further validation in defined patient subgroups.

**TABLE 6:** Small molecule protein inhibitors targeting the HER2^+^Undergoing clinical studies in colorectal cancer.

SMKIs	NCT number	Targets	Phase	Subject (n)	Treatment	ORR	OS	PFS	Evidence level	Interpretation/Comment
Lapatinib	NCT03225937 (Sartore-Bianchi et al., 2016)	EGFR/HER2	II	HER2+ mCRC (n = 27)	Trastuzumab + Lapatinib	28%	10 m	4.7 m	Moderate clinical evidence	Prospective HER2-selected phase II evidence supports proof of concept, but the small cohort and dual-antibody/TKI design limit broad extrapolation.
Neratinib	NCT01960023 (Jacobs et al., 2021)	HER1/HER2/HER4	Ib	WT (KRAS, NRAS,BRAF,PIK3CA)mCRC (n = 21)	Neratinib + Cetuximab	NA	NA	NA	Exploratory	Early-phase study without reported efficacy outcomes in the uploaded table; conclusions about CRC benefit cannot be made.
Tucatinib	NCT03043313 (Strickler et al., 2023)	HER2	II	HER2+/ RAS WT mCRC (n = 117)	Trastuzumab + Tucatinib	38.1%	23.9 m	8.1 m	Relatively mature evidence	Biomarker-selected phase II evidence demonstrates meaningful activity in HER2-positive/RAS-WT mCRC.
Pyrotinib	NCT05350917	HER1/HER2/HER4	II	HER2-positive or mutated advanced colorectal cancer	Tislelizumab (PD1 inhibitor) +DisitamabVedotin (ADC)+ Pyrotinib	NA	NA	NA	Exploratory	No efficacy outcomes are available; interpretation should await mature prospective results.
Pyrotinib	NCT04380012 (Fu et al., 2023)	HER1/HER2/HER4	II	HER2+ mCRC (n = 20) RAS WT (n = 12)	Trastuzumab + Pyrotinib	22.2% 33.3%	NA	3.4 m 4.3 m	Preliminary clinical signal	Small HER2-selected cohort suggests activity, especially in RAS-WT patients, but sample size and short PFS limit certainty.
DS-8201	NCT03384940 (Yoshino et al., 2023)	HER2	II	HER2+ mCRC	DS-8201	45.3%	NA	6.9 m	Relatively mature evidence	HER2-selected phase II evidence shows strong activity.

DS-8201, is an antibody-drug conjugate rather than an SMKI, and is retained as a HER2-pathway comparator.

Integrated interpretation. HER2-directed strategies show clearer activity than many other exploratory SMKI, approaches, but only in carefully selected HER2-positive, often RAS-wild-type CRC., the main limitation is not lack of target validity but limited applicability, because HER2 alterations are uncommon and biologically heterogeneous. Differences in HER2 amplification level, intratumoral heterogeneity, coexisting RAS/BRAF/PIK3CA, alterations, and prior anti-EGFR, exposure may influence response.

#### 5.3.2 MET

The mesenchymal-to-epithelial transition factor (MET), also referred to as cellular-mesenchymal-to-epithelial transition factor (c-MET) or hepatocyte growth factor receptor (HGFR), is a receptor tyrosine kinase expressed at the cell surface that has been implicated as an oncogenic driver in multiple solid tumors (Kim et al., 2016; Sun Z. et al., 2018; Tang et al., 2021). Its ligand, hepatocyte growth factor (HGF), binds MET and induces receptor dimerization and tyrosine phosphorylation (predominantly at Y1234/Y1235), thereby activating multiple downstream signaling cascades, including the PAM, MAPK, and STAT3 pathways (Joosten et al., 2019). Aberrant activation of MET is considered an important therapeutic target in CRC (Parizadeh et al., 2019). Studies indicate that MET amplification or HGF overexpression is associated with primary or acquired resistance to EGFR inhibitors in CRC patients (Liska et al., 2011; Jones et al., 2024). A preclinical study showed that addition of exogenous HGF to cetuximab-treated CRC cells induces MET phosphorylation without markedly affecting phosphorylation of EGFR or ERBB3; this reactivates MAPK and PAM signaling and restores cellular proliferative capacity. These findings suggest that HGF-mediated activation of the MET pathway represents a key mechanism of resistance to EGFR-targeted therapy in CRC (Liska et al., 2011).

SMKIs targeting MET are classified by binding conformation and selectivity into two categories: Type I selective inhibitors and Type II multi-target inhibitors. Type I inhibitors bind the active conformation of the MET kinase and tend to exhibit higher selectivity; representative agents include capmatinib (INC280), tepotinib, and savolitinib (Gan et al., 2019; Mauri et al., 2022). These oral agents display high affinity for MET and relatively low off-target activity. Type II inhibitors stabilize the inactive kinase conformation and frequently act on multiple signaling axes. Representative Type II agents include tivantinib (ARQ-197), cabozantinib, crizotinib, and foretinib (Eng et al., 2016). In addition to MET inhibition, these compounds often target VEGFR, AXL, RET, or ALK, enabling concurrent suppression of tumor proliferation, angiogenesis, and resistance pathways.

MET inhibitors are under active investigation, but their application in MET-amplified mCRC remains relatively limited; clinical trials of MET inhibitors in CRC are summarized in Table 7. Studies have shown that savolitinib, a selective MET TKI, can rapidly induce partial responses (PR) in MET-amplified CRC patients (Jia et al., 2024). Early studies have reported that combinations such as tivantinib with cetuximab and irinotecan may prolong survival in resistant KRAS wild-type metastatic CRC (Eng et al., 2016). However, these data remain limited and require confirmation in larger biomarker-defined studies. Because MET alterations can confer resistance, exploration of dual-targeting combination strategies is critical; indeed, tepotinib combined with osimertinib has shown preliminary activity in MET-amplified, *EGFR*-mutant non-small-cell lung cancer (Wu Y.-L. et al., 2024). This strategy offers a new rationale for combination therapy exploration in CRC, but further *in vitro* and preclinical validation of such combinations in CRC is required.

**TABLE 7:** Small molecule protein inhibitors targeting the MET undergoing clinical studies in colorectal cancer.

SMKIs	NCT number	Targets	Phase	Subject (n)	Treatment	ORR	OS	PFS	Evidence level	Interpretation/Comment
Tivantinib	NCT01075048 (Eng et al., 2016)	MET	I/II	wtKRAS mCRC (n = 60)	Tivantinib + Cetuximab + Irinotecan	45%	19.8 m	8.3 m	Exploratory	Early combination study showed activity, but benefit is difficult to attribute to MET inhibition because patients were not clearly MET-selected and chemotherapy/EGFR blockade confounded interpretation.
Tivantinib	NCT01892527 (Rimassa et al., 2019)	MET	II	wtKRAS mCRC (n = 41)	Tivantinib + Cetuximab	9.8%	9.2 m	2.6 m	Limited/ negative evidence	Phase II data show limited activity despite KRAS-wild-type selection; lack of MET amplification or HGF/MET biomarker enrichment may explain weak benefit.
Cabozantinib	NCT03542877 (Scott et al., 2022)	MET	II	mCRC (n = 40)	Cabozantinib	3%	8.3 m	3.0 m	Limited/ negative evidence	Single-agent multikinase inhibition showed low ORR and modest PFS, suggesting limited unselected activity and need for molecularly defined MET-driven populations.
Cabozantinib	NCT03539822 (Saeed et al., 2024)	MET	II	pMMR/MSS mCRC (n = 29)	Cabozantinib + Durvalumab	44.8%	NA	9.1 m	Preliminary clinical signal	Combination showed encouraging activity in a small pMMR/MSS cohort, but single-arm design and uncertain predictive biomarkers require prospective validation.

Integrated interpretation. MET-targeted approaches remain investigational in CRC., Although HGF-MET, activation can mediate resistance to EGFR-directed therapy and reactivate MAPK/PAM, signaling, clinical studies have often enrolled KRAS-wild-type or unselected patients rather than prospectively defined MET-amplified or HGF/MET-activated subgroups. Low ORR, in several studies suggests that MET, inhibition alone is insufficient for most CRC, patients. Future trials should require standardized MET, biomarker definitions, clarify the role of MET, in acquired resistance, and test rational combinations in molecularly selected cohorts.

#### 5.3.3 ALK

ALK is a receptor-type tyrosine kinase that belongs to the insulin receptor superfamily. Gene fusions, amplifications, or mutations of ALK have been identified across multiple tumor types (such as non–small-cell lung cancer, anaplastic large-cell lymphoma, neuroblastoma) and have been confirmed as oncogenic drivers (Morris et al., 1994; Soda et al., 2007). Upon activation, ALK undergoes autophosphorylation and triggers downstream signaling cascades, principally MAPK, PAM, and JAK/STAT3 pathways, thereby promoting tumor cell proliferation, survival, and immune evasion (Bai et al., 2000; Hallberg and Palmer, 2016). The incidence of ALK aberrations in CRC is low (approximately 0.05%–2.5%); however, ALK fusions or amplifications are associated with more aggressive phenotypes and poorer clinical prognosis, particularly in advanced mCRC (Wang Y. et al., 2022).

Several ALK-targeting SMKIs have been developed and clinically deployed, including first-generation crizotinib, second-generation alectinib and ceritinib, and third-generation lorlatinib (Hu et al., 2025). These agents competitively bind the ATP-binding pocket of the ALK kinase domain, blocking activation of downstream pathways and thereby inhibiting tumor cell proliferation and survival.

Although ALK fusions are uncommon in CRC, case reports have documented marked clinical response in mCRC patients harboring echinoderm microtubule-associated protein-like 4 (EML4)–ALK fusions treated with crizotinib, alectinib, or lorlatinib, with substantial tumor shrinkage and disease stabilization lasting several months, thereby supporting the potential utility of ALK-targeted therapy in CRC (He et al., 2021). These observations support the biological plausibility of ALK-targeted therapy in rare ALK-altered CRC. Nevertheless, the evidence remains preliminary and is largely derived from case reports or small series rather than prospective CRC-specific trials. Therefore, ALK inhibitors should be considered only for molecularly selected patients, and their clinical role in CRC requires validation in larger studies (Poei et al., 2024).

#### 5.3.4 Other emerging targets

Several rare but targetable molecular alterations in CRC merit attention. NTRK genes belong to RTKs, which are activated by neurotrophins and trigger downstream cascades such as MAPK, PAM, and PLC-γ, promoting aberrant proliferation, migration, invasion, and survival that contribute to tumorigenesis (Manea et al., 2022). NTRK gene fusions are exceedingly rare in CRC, with a reported prevalence below 1% (Lasota et al., 2020). TRK inhibitors such as larotrectinib and entrectinib have demonstrated marked efficacy in NTRK-fusion–positive solid tumors, with some patients achieving pronounced tumor regression or even complete responses (Doebele et al., 2020; Hong et al., 2020). RET fusions, well documented in NSCLC and thyroid cancer, are rare in CRC, occurring at an estimated frequency of ∼0.2% (Nagasaka et al., 2023). However, these targets occur in only a small fraction of CRC patients, and the efficacy of corresponding SMKIs should be evaluated in molecularly selected populations rather than extrapolated to unselected CRC. Thus, rare fusion-directed SMKIs may offer precision-medicine options for selected patients, but their CRC-specific clinical value remains dependent on biomarker confirmation and further validation.

### 5.4 Negative, marginal, and discontinued SMKIs-related programs in CRC

A balanced interpretation of SMKIs development in CRC should include not only successful or promising studies but also negative, marginal, and discontinued programs. Several small-molecule targeted agents showed strong biological rationale or early activity, but failed to establish meaningful clinical benefit in phase III trials or were not further developed as standard CRC therapies. Common limitations included lack of OS improvement, absent or marginal PFS/ORR benefit, toxicity-related dose reduction or treatment discontinuation, insufficient biomarker selection, and failure of exploratory signals to translate into durable clinical outcomes. Representative examples are summarized in Table 8. Overall, these trials indicate that biological rationale alone does not guarantee clinical benefit. Some programs were discontinued because of potential futility, limited feasibility, or toxicity-related concerns.

**TABLE 8:** Representative negative, marginal, or discontinued SMKIs-related clinical programs in CRC.

SMKIs	NCT number	Targets	Phase	Subject	Treatment	Result status	Main result/Reason
Brivanib	NCT00640471 (Siu et al., 2013)	VEGFR/ FGFR	III	Chemotherapy-refractory KRAS WT mCRC	Brivanib + cetuximab vs. cetuximab	No meaningful OS benefit	Toxicity increased and dose intensity was reduced
Cediranib	NCT00384176 (Hoff et al., 2012)	VEGFR1–3	III	mCRC	Cediranib + mFOLFOX6 vs. bevacizumab + mFOLFOX6	No meaningful clinical advantage	Patient-reported outcomes were less favorable
Cediranib	NCT00399035 (Schmoll et al., 2012)	VEGFR1–3	III	mCRC	Cediranib + FOLFOX/ CAPOX vs. chemotherapy	Marginal PFS signal, no OS benefit	Clinical value was limited.
Vatalanib	NCT00056459 (Hecht et al., 2011)	VEGFR	III	mCRC	Vatalanib + FOLFOX4 vs. placebo + FOLFOX4	No meaningful clinical benefit	No statistically significant improvement in PFS, OS, or ORR
Sunitinib	NCT00457691 (Carrato et al., 2013)	VEGFR	III	mCRC	Sunitinib + FOLFIRI vs. placebo + FOLFIRI	Terminated for futility/ poor tolerability	Potential futility; PFS was not superior and toxicity was increase
Nintedanib	NCT02149108 (Van Cutsem et al., 2018)	VEGFR/ FGFR	III	Refractory mCRC	Nintedanib + best supportive care vs. placebo + best supportive care	Marginal PFS signal, no OS benefit	Did not meet both co-primary endpoints; PFS improved modestly, but OS was not improved and objective responses were minimal
Napabucasin	NCT01830621 (Jonker et al., 2018)	STAT3/ β-catenin	III	Refractory advanced CRC	Napabucasin vs. placebo	No OS benefit in overall population	OS was not improved in the overall population; pSTAT3-positive subgroup signal was exploratory
Napabucasin	NCT02753127 (Shah et al., 2023)	STAT3/ β-catenin	III	Previously treated mCRC	Napabucasin + FOLFIRI ± bevacizumab	No meaningful clinical benefit	Failed to improve OS in both the overall population and the pSTAT3-positive subgroup

Across the clinical studies summarized in Tables 2–8, the objective response rates of numerous SMKIs-based regimens remain relatively low, with improvements in overall survival often being modest or absent. Several factors may account for these observations. Firstly, many multikinase inhibitors, particularly anti-angiogenic agents, primarily exert cytostatic effects rather than directly cytotoxic ones. Consequently, their efficacy is more frequently manifested through disease stabilization or modest prolongation of PFS rather than significant tumor shrinkage (Grothey et al., 2013). Secondly, numerous trials have been conducted in heavily pretreated patients with refractory mCRC, where high tumor burden, liver metastases, impaired performance status, and accumulated treatment-related toxicities may diminish the likelihood of a durable response and attenuate OS benefits. Thirdly, inadequate molecular selection may dilute treatment effects, as many kinase-dependent vulnerabilities are confined to small biomarker-defined subgroups rather than unselected CRC populations (Seidman et al., 2020). Lastly, pathway redundancy and compensatory feedback activation can swiftly restore downstream signaling following single-node inhibition, thereby limiting the durability of the response. Finally, trial design factors, including small sample size, single-arm design, heterogeneous prior therapies, insufficient pharmacodynamic confirmation, dose reduction, treatment discontinuation, crossover, and post-progression therapy, may further influence the interpretation of ORR, PFS, and OS. These findings indicate that future SMKI trials should incorporate biomarker-defined enrollment, toxicity-aware dosing, rational combination design, and clinically meaningful endpoints to better determine whether mechanistic rationale can translate into durable clinical benefit.

## 6 Mechanisms of resistance and counterstrategies

### 6.1 Primary resistance and acquired resistance

**FIGURE 3:**
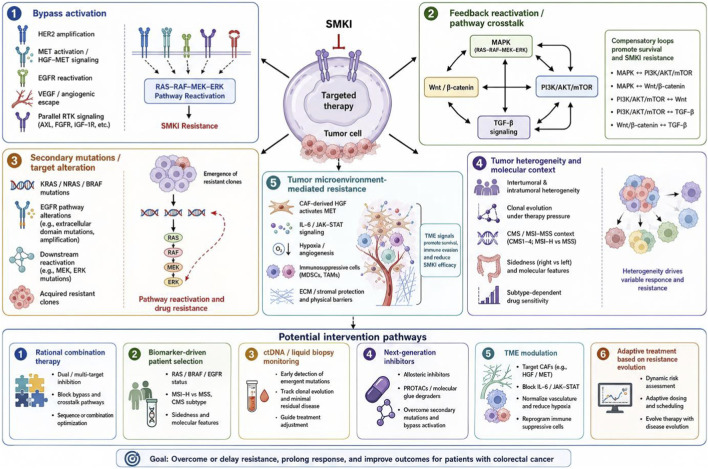
Resistance mechanisms and potential intervention pathways of SMKIs in colorectal cancer. The schematic summarizes major mechanisms limiting SMKI efficacy in CRC, including bypass pathway activation, feedback reactivation and pathway crosstalk, secondary target alterations and downstream reactivation, tumor heterogeneity and molecular context, and tumor microenvironment-mediated resistance. Potential counterstrategies include rational combination therapy, biomarker-driven patient selection, ctDNA/liquid-biopsy monitoring, next-generation inhibitors such as allosteric inhibitors and PROTACs, tumor microenvironment modulation, and adaptive treatment adjustment.

Drug resistance remains a fundamental challenge, typically classified as either primary resistance or acquired resistance. Primary resistance refers to the intrinsic insensitivity of tumor cells to SMKIs at the initiation of therapy. A classic example is the presence of KRAS mutations, especially the *KRAS G12C* variant, which confers innate resistance to EGFR-targeted therapies, thereby precluding clinical benefit from EGFR inhibitors in these patients (Yaeger et al., 2018). Acquired resistance develops during treatment after an initial period of disease control. It may result from secondary alterations in the drug target, reactivation of downstream signaling, activation of bypass pathways, or clonal selection of resistant subpopulations. For instance, in tumors harboring ALK fusion genes, secondary mutations such as C1156Y or L1198F may arise, which reduce the binding affinity of ALK inhibitors and mediate resistance (Shaw et al., 2016). Similarly, in *BRAF V600E*-mutant CRC, monotherapy with BRAF inhibitors can initially suppress MAPK signaling but eventually induces compensatory activation of EGFR, leading to MAPK pathway reactivation and resistance (Corcoran et al., 2012). The major resistance mechanisms and potential intervention pathways of SMKIs in CRC are summarized in Figure 3.

### 6.2 Tumor heterogeneity and molecular context

Tumor heterogeneity and molecular background also significantly increase the difficulty of achieving a response to SMKI-targeted therapy for colorectal cancer. CRC is not a single biological entity; instead, treatment sensitivity is strongly influenced by microsatellite instability status, distinct molecular subtypes, primary tumor location, and coexisting molecular alterations.

First, colorectal cancer with high microsatellite instability or deficient mismatch repair (MSI-H/dMMR) represents a distinct molecular and immunological subgroup. MSI-H tumors are generally characterized by a high mutational burden, enrichment of immune-activation features, and frequently overlap with CMS1 tumors. In addition, MSI-H status is frequently associated with *BRAF V600E* mutation and MLH1 promoter methylation (Gatalica et al., 2016; Adar et al., 2017), suggesting that the epigenetic background and MAPK pathway alterations may jointly influence the therapeutic efficacy of SMKIs. Therefore, in MSI-H/dMMR CRC, the effects of SMKIs should be interpreted in the context of immune responsiveness and coexisting molecular alterations, rather than independently of MSI status.

Second, CMS classification refines the molecular heterogeneity of CRC at the transcriptomic level (Dienstmann et al., 2017). CMS1 is characterized by MSI and immune activation; CMS2 by epithelial differentiation with WNT/MYC activation; CMS3 by metabolic dysregulation and frequent KRAS alterations; and CMS4 by mesenchymal features, TGF-β activation, stromal invasion, angiogenesis, and an immunosuppressive microenvironment (Schmitt and Greten, 2021). These biological differences may influence sensitivity to kinase-targeted therapy by altering pathway dependency, stromal signaling, and resistance mechanisms. However, CMS-guided targeted therapy remains investigational and has not yet been established as a routine criterion for SMKI selection in clinical practice.

Third, primary tumor sidedness is also a clinically relevant surrogate for molecular context. Left-sided RAS wild-type CRC generally derives greater benefit from EGFR-directed therapy, whereas right-sided tumors are more frequently enriched for MSI-H status, BRAF mutation, CIMP-high phenotype, and poorer response to EGFR-targeted approaches (Tejpar et al., 2017; Baran et al., 2018). Although anti-EGFR antibodies are not SMKIs, these data remain relevant to SMKIs development because they reflect sidedness-dependent EGFR/MAPK pathway dependency and feedback biology. Therefore, future SMKI trials should incorporate MSI status, CMS subtype, tumor sidedness, and coexisting genomic alterations to avoid dilution of therapeutic effects in molecularly unselected populations.

### 6.3 Key signaling-pathway alterations associated with resistance

Although resistance arises through genetically and epigenetically heterogeneous mechanisms, the molecular alterations implicated predominantly converge on core signaling axes (Li et al., 2020). The pathways establish interconnected signaling networks, where inhibiting one node may cause compensatory activation of upstream receptors, parallel pathways, or downstream effectors.

#### 6.3.1 MAPK pathway and PAM pathway

The MAPK and PAM pathways constitute two core oncogenic signaling axes in CRC that are frequently co-activated and interact with one another (Mendoza et al., 2011). Following ligand activation of EGFR, distinct adaptor proteins concurrently initiate both MAPK and PAM signaling, driving tumor cell proliferation and survival. In addition, RAS proteins can directly bind and activate the p110 catalytic subunit of PI3K, thereby amplifying cross-pathway signaling. Certain downstream effectors act as integrative nodes for both pathways; for example, 4E-BP1 is a key mediator through which AKT and ERK jointly regulate protein synthesis (Hsieh et al., 2012). ERK/RSK- and AKT-mediated regulation of the TSC–mTORC1 axis further illustrates how these pathways converge on common downstream effectors (Mendoza et al., 2011). These mechanisms provide a biological rationale for combined pathway inhibition, but they do not by themselves establish clinical efficacy. Notably, co-mutations such as *PIK3CA* with KRAS or BRAF, and the co-occurrence of *PTEN* loss with BRAF mutations, indicate that tumor cells may abrogate dependence on a single signaling axis by concurrently altering key genes in both pathways. Therefore, when only one pathway is targeted for inhibition, the compensatory activation of the other pathway often allows tumor cells to continue surviving, which is also an important reason for drug resistance. To address this issue, preclinical studies have proposed concurrent inhibition of MAPK and PAM pathways to more effectively suppress downstream mTOR signaling and achieve greater antitumor efficacy (Corcoran et al., 2012). However, such dual-pathway strategies should be interpreted cautiously because clinical translation has been limited by toxicity, incomplete pathway suppression at tolerable doses, tumor heterogeneity, and the lack of validated predictive biomarkers. Vito Amodio et al. (Amodio et al., 2020) validated in *KRAS G12C* CRC models that adaptive reactivation of EGFR is a principal mechanism of resistance to *KRAS G12C* inhibition, and that combined EGFR blockade can reverse this resistance and significantly enhance the antitumor efficacy of *KRAS G12C* inhibitors *in vivo*. Nevertheless, these benefits remain confined to a small molecularly defined subgroup, and resistance can still emerge. Therefore, vertical or parallel pathway blockade should be regarded as a rational, biomarker-dependent strategy rather than a broadly established solution for CRC. Future combination approaches should incorporate molecular selection, toxicity management, and prospective validation to determine whether mechanistic synergy can translate into durable clinical benefit.

#### 6.3.2 MAPK pathway and the Wnt/β-catenin pathway

Wnt/β-catenin and MAPK pathways interact via EGFR/KRAS nodes, transcriptional cross-regulation, and mutational cooperation, forming a networked interplay that jointly promotes CRC progression and therapeutic resistance (Russo et al., 2018). Interactions commonly arise through the following mechanisms: firstly, MAPK signaling upregulates Wnt activity—upon EGFR activation, promotion of AXIN1 phosphorylation leads to disassembly of the β-catenin destruction complex, thereby enhancing β-catenin stability (Hu and Li, 2010). Secondly, KRAS mutations drive dual-pathway activation: KRAS mutations not only activate the MAPK cascade but also elicit collateral effects, as KRAS mutants can phosphorylate SMAD2/3, thereby impeding TGF-β tumor-suppressive activity and indirectly promoting Wnt pathway activation (Cancer Genome Atlas Network, 2012). This bidirectional activation mechanism causes MAPK and Wnt pathways to interact in CRC, making isolated blockade of either pathway difficult. Resistance to MAPK inhibitors is frequently accompanied by compensatory activation of the Wnt pathway. Conversely, resistance to Wnt inhibitors can induce expansion of KRAS/BRAF-mutant clones. In addition, studies indicate that HSPA8 activates Wnt/β-catenin signaling and, via chaperone-mediated autophagy (CMA)-mediated degradation of CAV1, promotes the progression of *BRAF V600E* colorectal cancer, which supports a mechanistic link between BRAF-driven MAPK signaling, Wnt activation, and tumor progression (Li et al., 2024a). Therefore, targeted combination interventions represent an effective strategy to overcome single-agent resistance (Zhan et al., 2019).

These findings offer a biological rationale for the concurrent targeting of Wnt/β-catenin and MAPK-related signaling pathways. Nonetheless, this rationale should not be misconstrued as evidence of clinically validated benefits. The majority of Wnt-targeted strategies remain in preclinical or early clinical stages, and direct inhibition of the Wnt pathway is complicated by the pathway’s complexity, its role in normal tissue homeostasis, associated toxicity, and the absence of validated predictive biomarkers. Consequently, interventions targeting both Wnt and MAPK pathways should be considered investigational and biomarker-dependent, rather than established therapeutic strategies in CRC. Future research should focus on determining whether specific molecular contexts, such as APC/KRAS co-alteration, *BRAF V600E* mutation status, or Wnt-high transcriptional states, can identify patients most likely to benefit. These studies should also rigorously assess the feasibility, toxicity, and durability of response associated with these interventions.

#### 6.3.3 PAM pathway and the Wnt/β-catenin pathway

The PAM and Wnt/β-catenin pathways cooperate through multilayered interactions to drive tumor progression; the specific mechanisms are as follows: (1) PAM enhances Wnt signaling: AKT phosphorylates and inactivates GSK3β, preventing β-catenin degradation, leading to its cytoplasmic accumulation and nuclear translocation to activate targets such as c-Myc and Cyclin D1 (Valvezan et al., 2012). Additionally, mTORC1 phosphorylates Dishevelled protein DVL1 to enhance Wnt receptor signaling, and mTORC1-mediated phosphorylation via S6K and 4E-BP1 can increase β-catenin expression (Faller et al., 2015). (2) Wnt feedback activates PAM: β-catenin/TCF complexes transcriptionally upregulate *PIK3CA*, directly activating PI3K/AKT signaling. These interactions provide a biological rationale for co-targeting PAM and Wnt signaling in selected molecular contexts.

The PAM and Wnt/β-catenin pathways exert significant compensatory feedback regulation that promotes tumor cell survival, limiting the efficacy of single agent targeted therapies and fostering resistance (Fleming-de-Moraes et al., 2022). Preclinical studies show that AKT inhibitors, by suppressing the PAM axis, may further activate the Wnt pathway to maintain tumor proliferation; conversely, Porcupine inhibitors can induce feedback activation of PI3K/AKT, forming bidirectional regulatory feedback loops. Notably, APC, *PIK3CA*, and KRAS mutations frequently co-occur in CRC, resulting in persistent activation of Wnt and PAM pathways and rendering single-pathway inhibition prone to failure (Deming et al., 2014). Furthermore, *PTEN* loss or AXIN1 mutations can exacerbate feedback activation between PAM and Wnt pathways, precipitating acquired resistance (Arqués et al., 2016). Dual PI3K/mTOR inhibition has shown antitumor activity in APC/*PIK3CA*-mutant preclinical CRC models, suggesting a potential vulnerability that requires clinical validation (Foley et al., 2017). Thus, the combined targeting of PAM and Wntt/β-catenin is still experimental and needs to be evaluated in biomarker-specific clinical trials, paying close attention to pathway feedback, molecular variability, toxicity, and sustained clinical outcomes.

#### 6.3.4 PAM pathway and the TGF-β pathway

The PAM and TGF-β/SMAD pathways exhibit both cooperative and antagonistic interactions in tumors. As a canonical tumor-suppressive pathway, TGF-β can mediate cell-cycle arrest and pro-apoptotic effects via SMAD signaling in early CRC. However, activated PAM signaling can weaken the inhibitory effect of the TGF-β/SMAD pathway through multiple mechanisms (Luo, 2017). Studies have found that AKT can phosphorylate the linker region of SMAD3, impairing its nuclear translocation and transcriptional activity, thereby weakening TGF-β downstream gene regulation. Conversely, TGF-β can induce PRL-3 upregulation and, via non-canonical routes, activate PI3K/AKT signaling to promote cell survival, EMT, and tumor invasiveness (Jiang et al., 2011). In CRC, PRL-3 has been identified as a direct regulatory target of TGF-β and can enhance cell survival and metastatic potential partly through AKT-related signaling (Kim et al., 2002). These observations support a biological rationale for considering the PAM/TGF-β interaction in CRC progression and resistance. Therapeutic strategies targeting both pathways remain largely investigational, and their translation is limited by pathway pleiotropy, molecular heterogeneity, compensatory signaling, and potential toxicity. Therefore, PAM–TGF-β co-targeting should be regarded as a hypothesis-driven strategy that requires biomarker-defined patient selection and prospective clinical validation before being considered a clinically proven SMKI-based approach in CRC.

#### 6.3.5 Wnt/β-catenin pathway and the TGF-β pathway

TGF-β can upregulate Wnt ligands (Wnt2/4/5a/7a/10a) and the co-receptor LRP5, activate TGF-β/SMAD signaling, and enhance nuclear accumulation and stability of β-catenin. Concurrently, SMAD proteins can interact with LEF1/TCF transcription factors to cooperatively activate both TGF-β and Wnt/β-catenin pathways (Tang et al., 2020). In approximately 50% of early adenomas and colorectal cancers, activating KRAS mutations phosphorylate the linker domain of SMAD2/3, impeding SMAD nuclear translocation and thereby suppressing their growth-regulatory functions (Kretzschmar et al., 1999). These data provide a mechanistic basis for exploring combined modulation of Wnt/β-catenin and TGF-β signaling. Nevertheless, this approach remains exploratory. Direct Wnt inhibition is challenging because of pathway complexity and potential toxicity in normal tissue homeostasis, while TGF-β blockade may produce context-dependent effects because of its dual tumor-suppressive and tumor-promoting roles. Therefore, Wnt–TGF-β co-targeting should be framed as an investigational strategy rather than an established therapeutic solution for CRC.

### 6.4 Tumor microenvironment

The tumor microenvironment (TME) is another major contributor to SMKI resistance. Resistance is driven not only by tumor cell-intrinsic signaling plasticity but also by stromal, immune, vascular, and extracellular matrix components that provide protective or compensatory signals. CAFs can secrete growth factors, cytokines, and exosomes that activate bypass signaling pathways in tumor cells. For example, CAF-derived HGF can activate the c-MET pathway and directly promote acquired resistance to anti-EGFR therapies (Straussman et al., 2012). In addition, recruitment and activation of myeloid-derived suppressor cells (MDSCs) can, via secretion of cytokines such as IL-6 and TGF-β, enhance immunosuppression, promote tumor stemness, and upregulate signaling axes including STAT3 and NF-κB, thereby indirectly diminishing SMKI efficacy (Kumar et al., 2016). These findings suggest that SMKI’s resistance in CRC cannot be fully explained by tumor genomic alterations alone. The TME may establish a microenvironment-driven resistance network that allows tumor cells to survive despite pharmacological inhibition of key signaling nodes. Therefore, future counterstrategies should consider not only tumor-cell targets but also stromal remodeling, immune suppression, and paracrine bypass signaling.

### 6.5 Epigenetic mechanisms

In recent years, extensive studies have demonstrated that epigenetic regulation plays a critical role in the establishment and maintenance of SMKI resistance. Aberrant DNA methylation can downregulate negative regulators of key signaling pathways (such as *PTEN* and *RASSF1A*), thereby relieving inhibition of PAM and MAPK axes and enhancing tumor cell resistance to SMKIs (Romero-Garcia et al., 2020). Additionally, noncoding RNAs further elaborate the resistance network by regulating drug transporters, signaling pathways, and apoptotic factors. Studies have confirmed that miR-21 and miR-135b downregulate the tumor suppressor *PTEN*, relieving negative regulation of the PAM pathway and thereby enhancing CRC cell resistance to SMKIs (Jiang et al., 2021); Furthermore, the lncRNA CCAT1 sponges miR-218 to upregulate VEGF expression, which not only promotes CRC cell migration and invasion but also, by enhancing VEGF/PI3K/AKT signaling and tumor angiogenesis, indirectly confers resistance to SMKIs and chemotherapeutic agents (Gu et al., 2020). The reversibility of epigenetic regulation offers novel avenues for reversing resistance and for combination therapies. Although HDAC or DNMT inhibitors may restore sensitivity to targeted therapy in preclinical models (Hu C. et al., 2021; Bajpai et al., 2024).

### 6.6 Designing new inhibitors to overcome drug resistance

In response to the resistance mechanisms described above, investigators are actively exploring design strategies for novel small-molecule inhibitors aimed at overcoming the resistance limitations of conventional agents. Yet, many of these strategies are still in the preclinical or early research phases for CRC, and their clinical importance requires additional confirmation.

First, PROTAC technology offers a mechanism distinct from conventional kinase inhibition. PROTAC molecules link a target-binding ligand to an E3 ubiquitin ligase recruiter, thereby inducing proteasomal degradation of the target protein rather than simply blocking catalytic activity. This approach may theoretically overcome resistance caused by target overexpression, certain kinase-domain mutations, or incomplete target inhibition. For example, dEALK1 can selectively degrade EML4-ALK and resistance-conferring ALK mutants, suggesting potential utility in ALK-rearranged tumors resistant to conventional ALK inhibitors (Li et al., 2024b). Nevertheless, the applicability of this strategy to CRC remains uncertain, particularly because of challenges related to tumor penetration, E3 ligase heterogeneity, degradation selectivity, pharmacokinetics, and off-target toxicity.

Second, allosteric inhibitors may overcome some limitations of ATP-competitive kinase inhibitors. DDO-6079 is a representative example that does not target the kinase ATP catalytic site but binds an allosteric pocket on the cochaperone CDC37, disrupting formation of the HSP90-CDC37-kinase complex, thereby selectively blocking maturation of multiple oncogenic kinases and reversing CDK6-mediated palbociclib resistance in CRC (Zhang L. et al., 2025). A related strategy is embodied by the small molecule DDO-5936 (Wang et al., 2019), which targets the key residue Glu47 on the HSP90 cochaperone to specifically disrupt HSP90-CDC37 interaction and selectively downregulate steady-state levels of a range of oncogenic kinases. The findings support the idea of using allosteric or chaperone-based strategies, but their clinical success and safety in CRC have yet to be confirmed.

Third, the development of bifunctional molecular inhibitors aims to target kinase activity while also affecting drug metabolism or transcriptional programs. Mustonen et al. (Mustonen et al., 2022) reported a class of anilino-benzo-cycloheptanone derivatives that function both as kinase inhibitors and as ligands of the pregnane X receptor (PXR), downregulating expression of the drug-metabolizing enzyme CYP3A4, thereby reducing *in vivo* drug clearance, increasing effective plasma exposure, and reversing resistant phenotypes. Although they are conceptually appealing, bifunctional molecules require careful pharmacological scrutiny because modifying drug-metabolizing enzymes might raise the chances of drug interactions or systemic toxicity.

Fourth, high-throughput screening and functional reporter systems have identified small molecules capable of simultaneously targeting multiple oncogenic pathways. Choi et al. used a TOP-Flash reporter screen to identify the dual inhibitor CPD0857, which simultaneously suppresses Wnt/β-catenin and MAPK signaling and exhibits potent inhibitory activity against KRAS-mutant CRC (Choi et al., 2020). Such multi-target drug designs hold promise for overcoming resistance in KRAS-driven CRC. Multi-target methods may offer solutions to pathway redundancy, but they also introduce worries about selectivity, toxicity limits at certain doses, and selecting patients based on biomarkers.

## 7 Exploration of combination therapy strategies

### 7.1 Combination with chemotherapeutic agents

**FIGURE 4:**
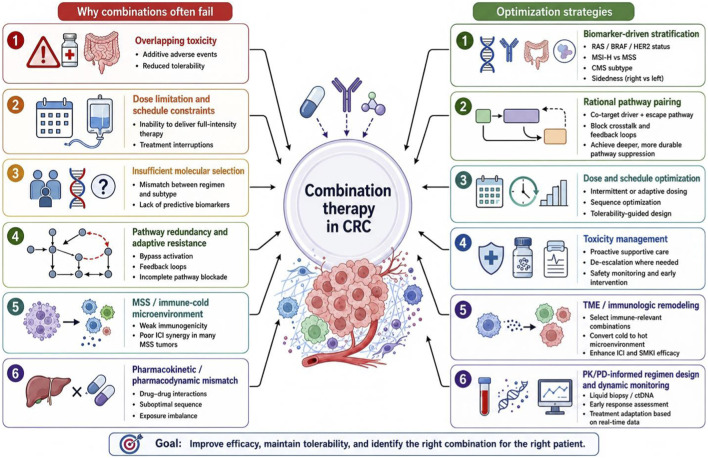
Causes of failure and optimization pathways for SMKI-based combination therapy. Major causes of failure include overlapping toxicity, dose and schedule constraints, insufficient molecular selection, pathway redundancy and adaptive resistance, an MSS/immunologically cold microenvironment, and pharmacokinetic/pharmacodynamic mismatch. Optimization strategies include biomarker-guided stratification, rational pathway pairing, dose and schedule optimization, proactive toxicity management, tumor microenvironment remodeling, and PK/PD-informed dynamic monitoring.

Combined application of SMKIs and chemotherapeutic agents often yields synergistic effects and is regarded as an important strategy in the management of CRC. The mechanisms underlying synergy between SMKIs and chemotherapeutic agents in CRC include: (1) SMKIs augment tumor-cell sensitivity to cytotoxics by inhibiting aberrantly activated signaling pathways; and (2) whereas chemotherapy induces DNA damage in tumor cells, concomitant SMKI administration can disrupt DNA-damage repair pathways, thereby promoting irreversible apoptotic death (Harata et al., 2023). The main reasons for failure of SMKI-based combination strategies and potential optimization approaches are illustrated in Figure 4.

Combination regimens of SMKIs with chemotherapeutic drugs are currently widely applied in clinical practice. In *BRAF V600E*–mutant mCRC, multi-targeted triplet regimens have shown notable efficacy: the phase-III BREAKWATER study reported that the triplet of encorafenib, cetuximab, and mFOLFOX6 achieved a confirmed ORR of 60.9% and prolonged median OS (follow-up) to 10.3 months, significantly outperforming historical standard therapies. This triplet regimen has the potential to become a new first-line standard for BRAF-mutant mCRC (Kopetz et al., 2025). Nevertheless, this benefit depends on *BRAF V600E* status, EGFR feedback biology, and the feasibility of multi-agent treatment. In contrast, several VEGFR TKIs–chemotherapy combinations, including cediranib-, vatalanib-, and sunitinib-based regimens, failed to establish standard roles in CRC because of absent or marginal survival benefit, toxicity, or treatment feasibility concerns (Schmoll et al., 2012). Another phase-III study, SUNLIGHT (Rais et al., 2024), demonstrated that in mCRC patients who had previously received multiple regimens or were unsuitable for standard first- or second-line therapy, a combination of the oral chemotherapeutic agent Lonsurf and bevacizumab significantly improved clinical outcomes. Results indicated that the combination arm achieved OS of 10.8 months and PFS of 5.6 months, representing a statistically significant improvement versus Lonsurf alone. These studies provide theoretical support for the combination of SKMI and chemotherapy.

### 7.2 Synergistic interactions with immune-checkpoint inhibitors

In recent years, combination therapies of SMKIs with ICIs have emerged as a major focus in oncology. SMKIs can promote immunogenic cell death (ICD) of tumor cells, facilitating tumor antigen release, enhancing antigen presentation, remodeling tumor vasculature, or reducing suppressive myeloid-cell activity, thereby increasing immune recognition of cancer cells (Wu B. et al., 2024). For example, MEK inhibitors may elicit tumor-intrinsic interferon responses that upregulate MHC class I and II molecule expression to enhance antigen presentation; concurrently, they can suppress immunosuppressive cell populations by reprogramming tumor-associated macrophages from an M2 to an antitumor M1 phenotype and by reducing infiltration of MDSCs and Tregs (Baumann et al., 2020). MEK inhibition may induce interferon-related programs and increase MHC expression, while VEGFR or CSF1R-related inhibition may remodel the tumor vasculature and myeloid compartment (Doleschel et al., 2021; Zhang and Brekken, 2022). These mechanisms provide a biological rationale for combining SMKIs with ICIs, particularly in immunologically “cold” proficient-MMR/microsatellite-stable (pMMR/MSS) CRC.

Based on these mechanisms, multiple clinical studies have recently investigated SMKI–ICI combinations and have yielded encouraging efficacy in subsets of refractory CRC patients. The phase-Ib REGONIVO trial (Fukuoka et al., 2020) reported that regorafenib combined with nivolumab (a PD-1 antibody) produced breakthrough efficacy in refractory pMMR/MSS mCRC, achieving an ORR of 33% (95% CI, 15.6%–55.3%) and a median PFS of 7.9 months, substantially exceeding historical controls. However, subsequent studies in broader or non-Japanese pMMR/MSS mCRC populations showed more modest activity, suggesting that efficacy may be influenced by patient selection, tumor burden, liver metastasis, prior therapy, dose intensity, and tumor immune contexture (Rossini et al., 2022). Additionally, a recently published phase-II trial (Tian et al., 2023) evaluated the efficacy of the BRAF inhibitor dabrafenib in combination with the MEK inhibitor trametinib and the PD-1 antibody spartalizumab. Results showed an ORR of approximately 25% for the combination (25% in MSS patients; overall 24.3%), meeting the primary endpoint, with some responses durable. Nevertheless, the study was small and non-randomized, and the clinical benefit of this approach requires validation in larger randomized trials before it can be considered established. Moreover, approximately 15% of CRC patients exhibit a deficiency of dMMR, a phenotype characterized by high tumor mutational burden that reduces the efficacy of standard chemotherapy, but this evidence remains limited by the small single-arm design and requires randomized validation (Parente et al., 2023). In contrast, immune checkpoint inhibitors are only applicable for the treatment of colorectal cancer in certain specific mutated molecules (such as MSI-H/dMMR). Therefore, the SMKIs-ICI combination therapy should be regarded as an emerging and biomarker-dependent strategy rather than a universal solution that can be widely used to overcome the resistance of colorectal cancer to immunotherapy. Future research should identify predictive biomarkers, optimize dosing and toxicity management, and demonstrate the achievement of sustained improvements in objective response rate, progression-free survival, overall survival, and quality of life.

### 7.3 Development of multi-targeted inhibitors

Design of multi-target inhibitors represents a prominent research focus in contemporary oncology. Conventional single-target inhibitors often exhibit limited efficacy due to tumor heterogeneity and signaling redundancy. By simultaneously targeting multiple key signaling nodes, multi-target inhibitors can more effectively suppress tumor growth and metastasis. Currently, the development of multi-target inhibitors has yielded preliminary successes and shows promising prospects. For example, LP-182 is an orally bioavailable multifunctional kinase inhibitor that concurrently targets PAM and MAPK pathways and demonstrated pronounced efficacy with low toxicity in myelofibrosis animal models (Kawazoe et al., 2024). Clinically used regorafenib is a prototypical multi-target receptor tyrosine kinase inhibitor that concurrently inhibits VEGFR, FGFR, PDGFR, KIT, RET, and other oncogenic nodes, and has conferred survival benefit in mCRC patients progressing after prior therapies (Grothey et al., 2013). Nintedanib, which simultaneously targets VEGFR, FGFR, and PDGFR, demonstrated PFS improvement in some studies when combined with FOLFIRI as second-line therapy for mCRC (Van Cutsem et al., 2018). Multi-target inhibitors have shown therapeutic potential in selected molecular subgroups of CRC, but their ability to improve efficacy or overcome resistance remains context-dependent and requires further clinical validation. Although pathway activation provides a biological rationale, mechanistic relevance does not necessarily translate into clinically meaningful benefit in CRC.

## 8 Challenges and future directions

Although SMKIs have achieved notable advances in CRC therapy, multiple challenges remain.

### 8.1 Complex resistance mechanisms and limited countermeasures

Mechanistically, resistance to SMKIs is complex. Targeting a single kinase or signaling pathway frequently induces compensatory activation of parallel or downstream pathways, resulting in incomplete pathway suppression and therapeutic escape (Yaeger et al., 2023). Consequently, monotherapy responses are often not durable. Vertical pathway blockade is a rational resistance-overcoming strategy, but its clinical relevance in CRC remains highly context-dependent. Current evidence mainly supports this approach in selected molecular subgroups, particularly BRAF V600E-mutant CRC, where combined BRAF and EGFR blockade, with or without MEK inhibition, can reduce feedback pathway reactivation. However, this strategy should not be interpreted as broadly established for unselected CRC, and issues such as toxicity, response durability, and acquired resistance remain unresolved.

### 8.2 Toxicity and patient tolerability

Although multi-target combination therapies can enhance efficacy, they also introduce challenges of cumulative toxicity. Overlapping toxicities from combined SMKIs may produce adverse events such as cardiovascular toxicity, rash, and diarrhea, necessitating strengthened toxicity management (Larkin et al., 2014; Du et al., 2021; Zhu et al., 2025). Balancing efficacy with toxicity is a critical issue that must be resolved before combination regimens can be adopted as first-line therapies. Toxicity management strategies include metabolomics-based prediction of adverse events and supportive-care innovations to improve patient adherence (Zhao Q. et al., 2022). Novel EGFR-antibody-functionalized polydopamine nanoparticles (PDA NPs), which electrostatically load FOLFIRI agents (denoted FOLFIRI-CTX@PDA NP), selectively target EGFR-overexpressing CRC cells to substantially reduce tumor cell viability while exhibiting low stromal cytotoxicity (Djermane et al., 2024). Such novel drug-delivery systems (e.g., nanoparticle carriers) enable co-encapsulation of multiple agents for tumor-targeted delivery, reducing systemic exposure and mitigating adverse events; this technology warrants further development in CRC combination strategies.

### 8.3 Biomarkers and personalized strategies

Tumor heterogeneity and biomarker development are central priorities. Multi-omics profiling and liquid-biopsy approaches can help characterize tumor microenvironment heterogeneity, monitor clonal evolution, and guide sequential targeted therapy (Djermane et al., 2024). In particular, dynamic circulating tumor DNA (ctDNA) analysis enables non-invasive detection of actionable and resistance-associated alterations, including *RAS/BRAF* mutations, ERBB2 amplification, ALK rearrangements, and EGFR-related bypass changes, thereby supporting molecular stratification and treatment adjustment in CRC (Disel et al., 2015; Lai et al., 2017; Li et al., 2019). Diaz et al. were the first to use serial ctDNA monitoring in mCRC patients treated with anti-EGFR antibodies and observed that secondary KRAS mutations emerged 5–10 months prior to radiographic progression, demonstrating real-time capture of acquired resistant clones (Diaz et al., 2012). These findings suggest that early monitoring of key genetic alterations by ctDNA may provide resistance alerts prior to radiographic progression and enable timely adjustment of therapeutic strategies. However, its routine use for guiding SMKI selection or treatment switching in CRC remains under development. Key challenges include assay sensitivity, tumor-shedding variability, standardized thresholds, timing of sampling, cost, and evidence that ctDNA-guided intervention improves survival or quality of life.

### 8.4 Integration of emerging technologies and translational medicine

**FIGURE 5:**
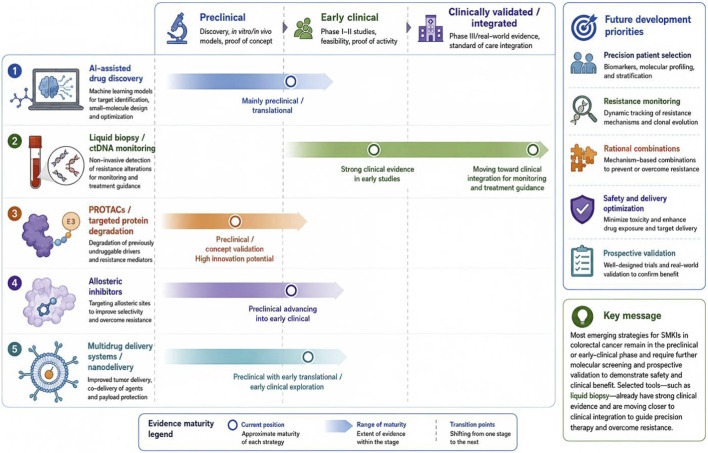
Evidence maturity of future directions for SMKIs in colorectal cancer. These schematic positions represent emerging strategies along an evidence continuum from preclinical to early clinical and clinically validated or integrated stages. Liquid biopsy/ctDNA monitoring currently shows relatively stronger clinical evidence, whereas AI-assisted drug discovery, PROTACs, allosteric inhibitors, and multidrug delivery systems remain largely preclinical or early translational. Future development should emphasize precision patient selection, resistance monitoring, rational combination strategies, safety and delivery optimization, and prospective validation.

Emerging technologies may help address several current limitations of SMKI development in CRC, including insufficient target selectivity, adaptive resistance, lack of predictive biomarkers, inadequate preclinical models, and cumulative toxicity. Rather than functioning as isolated tools, multi-omics profiling, AI-assisted drug discovery, patient-derived models, liquid biopsy, molecular imaging, and adaptive trial design can be integrated into a translational “discover–validate–monitor–adapt” workflow. The evidence, maturity, and translational status of these emerging strategies are summarized in Figure 5.

At the discovery stage, multi-omics profiling can identify molecular dependencies, resistance-associated alterations, and pathway crosstalk in distinct CRC subgroups. AI-assisted platforms may then integrate genomic, transcriptomic, proteomic, structural, and pharmacological data to identify candidate targets, predict drug–target interactions, optimize molecular structures, and screen compounds with improved selectivity (Dayton et al., 2023; Ren et al., 2025; Zhang K. et al., 2025). AI–assisted drug discovery has been applied to design highly selective kinase inhibitors such as the TNIK inhibitor INS018_055, which is currently undergoing clinical evaluation. Nevertheless, AI-generated compounds still require rigorous experimental validation, pharmacokinetic optimization, toxicity assessment, and prospective clinical testing before their therapeutic relevance in CRC can be established.

At the validation stage, patient-derived organoids and xenograft models may help evaluate SMKI efficacy, combination feasibility, and resistance mechanisms in biomarker-defined CRC models (Hidalgo et al., 2014). Materials-engineered multidrug delivery systems may further support pharmacological optimization by enabling co-delivery or sequential release of multiple small molecules, with the aim of improving local drug exposure and coordinating inhibition of complementary resistance pathways. The concept has been explored as a potential future scenario for multi-targeted cancer therapies and has been proposed within the framework of CRC multidrug strategies to address multiple resistance mechanisms (Navarro et al., 2024; Puzzo et al., 2025).

During clinical translation, serial ctDNA analysis and molecular imaging may support dynamic monitoring of clonal evolution, target expression, and emerging resistance. Jin et al.developed a near-infrared II fluorescent probe that concurrently recognizes EGFR and c-Met, and successfully visualized primary CRC lesions and metastatic lymph nodes in murine models (Jin et al., 2025). These tools may facilitate biomarker-enriched and adaptive trial designs, but their routine clinical use remains limited by assay standardization, model heterogeneity, sensitivity, specificity, cost, toxicity evaluation, and the need for prospective validation.

### 8.5 Clinical trials and strategy optimization

Future clinical trials should incorporate these emerging technologies into drug-development strategies in a stepwise manner. First, multi-omics and ctDNA profiling should be used to define biomarker-enriched cohorts rather than enrolling broadly unselected CRC populations. Second, AI-assisted modeling and patient-derived organoids may help prioritize rational combinations before clinical testing. Third, serial ctDNA and molecular imaging may be integrated as pharmacodynamic and resistance-monitoring tools to determine whether treatment failure results from inadequate target inhibition, pathway bypass activation, or clonal selection. Finally, adaptive basket or umbrella trials may allow dynamic refinement of treatment arms according to molecular response and emerging resistance patterns. Such designs may improve the efficiency of SMKI development, but they require standardized assays, rigorous external validation, toxicity-aware dosing, and clinically meaningful endpoints.

## 9 Conclusion

SMKIs have broadened treatment options for molecularly selected patients with advanced colorectal cancer, with clear clinical benefit demonstrated in specific molecular subtypes. However, their overall efficacy remains constrained by adaptive resistance, pathway redundancy, tumor heterogeneity, toxicity, and limited predictive biomarkers. Most emerging strategies still require rigorous molecular screening and prospective validation. Overall, SMKI-based therapy in CRC remains an evolving, subtype-dependent field that requires robust molecular stratification and clinically meaningful endpoints to achieve more durable and safer benefits.
